# GRACE-OFF: A Machine-Learned Interatomic Potential for Organic Liquids Using the GRACE Architecture

**DOI:** 10.1021/acs.jctc.6c01169

**Published:** 2026-09-03

**Authors:** Anna Katharina Picha, Johannes Karwounopoulos, Linus C. Erhard, Stefan Boresch, Esther Heid

**Affiliations:** † Faculty of Chemistry, Institute of Computational Biological Chemistry, 31285 University of Vienna, 1090 Vienna, Austria; ‡ Vienna Doctoral School of Chemistry (DosChem), 27258 University of Vienna, 1090 Vienna, Austria; § Institute of Materials Chemistry, 27259 TU Wien, A-1060 Vienna, Austria; ∥ Interdisciplinary Centre for Advanced Materials Simulation (ICAMS), Ruhr-University Bochum, 44780 Bochum, Germany

## Abstract

Machine-learned interatomic potentials (MLIPs) have become an increasingly important tool for molecular dynamics (MD) simulations, enabling near quantum-mechanical accuracy at significantly reduced computational cost. Recent studies indicate that the Graph Atomic Cluster Expansion (GRACE) neural network architecture delivers strong performance in materials chemistry. In this work, we assess the GRACE architecture for the prediction of potential energy surfaces for organic molecules and introduce GRACE-OFF (GRACE Organic Force Field). GRACE models of varying depth (one-layer and two-layer) and size (small, medium, large) are trained on the SPICE v2.0 data set. We validate the resulting models using a variety of benchmarks. These include single-point energy and force predictions, torsional energy profiles, condensed phase properties of organic liquids and water (thermodynamic properties, self-diffusion coefficients, radial distribution functions, and temperature-dependent water density), as well as the stability of biomolecular MD simulations for gas-phase Ala_15_ and solvated crambin. For the single-molecule benchmarks (single point energies and forces, torsional energy profiles), the one-layer models showed only mediocre performance, whereas the two-layer models outperformed the MACE-OFF models to which we compare. For the condensed phase properties, the two-layer models gave consistently better results than the MACE-OFF family of MLIPs. For water and hexane, the GRACE-OFF models also beat the much more expensive small UMA/OMol25 (S) model. The two-layer GRACE-OFF models accurately reproduce experimental water radial distribution functions and predict water densities in close agreement with experimental data over a temperature range from 270 to 330 K. Benchmarks demonstrate that GRACE-OFF achieves higher MD performance than comparable MACE-OFF models in both single and double precision. This establishes GRACE-OFF as an accurate and computationally efficient foundation potential for routine simulations of organic liquids and biomolecular systems.

## Graphical abstract

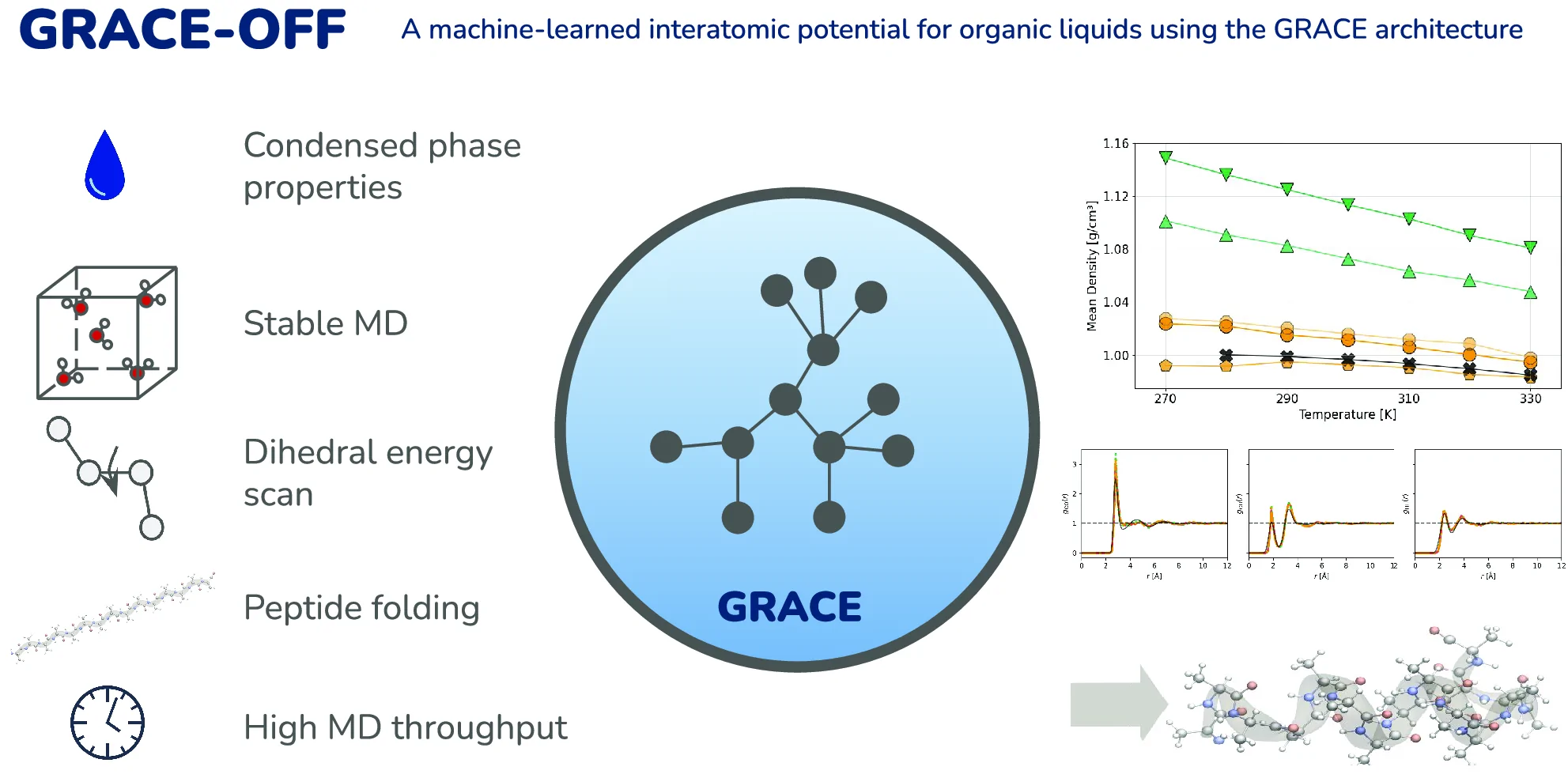

## 1 Introduction

The development of machine-learned interatomic potentials (MLIP), often also referred to as neural network potentials or quantum machine-learned potentials, has accelerated rapidly in recent years.^1–3^ Trained on high-quality quantum-mechanical (QM) reference data, these models can reproduce intra- and intermolecular interactions with an accuracy approaching the underlying QM methods, while requiring only a fraction of the computational effort. Numerous studies demonstrate that MLIPs can be developed for applications ranging from materials science to organic molecular systems, for example, for conformer sampling,^4^ prediction of molecular properties,^5^ phase transitions,^6^ and even free-energy simulations.^7–12^ Their computational efficiency is sufficient to enable molecular dynamics (MD) simulations of small- and medium-sized systems, such as water boxes or solute–solvent environments; simulations of proteins in aqueous solution have also been carried out, albeit at considerable computational cost.^13^ Various architectures have been proposed for MLIPs, offering a broad spectrum of complexity, accuracy, and computational cost.^14^ Notable examples include SchNet,^15^ ANI,^16^ DimeNet,^17^ SpookyNet,^18^ NequIP,^19^ Allegro,^20^ FENNIX,^21^ MACE^22^ and AIMNet2,^23^ among others.

MLIPs vary not only in their underlying architectures,^14^ but also in the strategies used for their training. When a model is (pre)­trained on broad data sets^24–28^ (not yet adapted to a particular task), it is commonly referred to as a foundation model.^13,29–31^ After training, the goal is typically to deploy a foundation model across a wide range of systems without additional QM calculations, or to further specialize it through fine-tuning for targeted applications. Due to their computational efficiency, foundation models are increasingly used as surrogates for high-level density functional theory (DFT) calculations during the development or refinement of force fields, as demonstrated, for instance, in ref 32. When system-specific training is done, MLIPs can also be used to describe reactive processes involving bond breaking and formation. This enables MD simulations of processes such as proton-transfer or catalytic reactions.^33,34^ Furthermore, some architectures are able to handle charges (see e.g., refs 23 or 35). However, at the time this work was initiated, most foundation models were limited to neutral species. Consequently, charged species and reactive processes are both outside the scope of this manuscript.

Apart from describing the complete system using an MLIP, other machine-learning (ML) methods are also becoming increasingly popular in computational chemistry. Some of these approaches combine ML with classical force fields (FFs).^7,8,36^ Rather than describing the entire system with an MLIP, these methods only model the chemically most relevant region using an MLIP, while the remainder of the system is represented by a classical FF. Other approaches employ ML to optimize FF parameters,^37,38^ and architectures such as FENNIX^21^ combine learned FF parameters with ML-based interaction models. Compared to classical FFs, the use of MLIPs and ML techniques in the context of MD is still comparatively recent. Systematic comparisons between MLIP architectures are therefore of particular importance, as such studies improve our understanding of the strengths and limitations of different architectures and help guide future methodological developments.

The atomic cluster expansion (ACE) framework has emerged as a powerful paradigm for constructing MLIP architectures.^39^ Two well-established architectures based on ACE are MACE^40^ and Graph Atomic Cluster Expansion (GRACE).^41^ The MACE architecture has been trained on a variety of data sets. For materials science, the MACE-MP-0 model family^29^ is widely used, while for organic molecules the MACE-Organic Force Field (MACE-OFF23/24) family provides pretrained models.^13^ GRACE has likewise been trained successfully on broad and chemically diverse data sets, though existing public models are currently limited to materials-science applications. Reported studies indicate that GRACE delivers strong performance in materials chemistry,^42–44^ underscoring its suitability for accurate energy and force prediction. In this work, we investigate whether GRACE can be trained effectively on an organic-system data set, specifically SPICE v2.0,^26^ and extend the architecture to a new family of foundation models. We deliberately keep the remaining training conditions as consistent as possible with those used for MACE-OFF to facilitate a more direct assessment of the effect of the underlying architecture. To systematically investigate the effects of size and depth on model performance, we consider several architectural variants. Drawing an analogy to the MACE-OFF family of MLIPs, we refer to GRACE models trained on SPICE as GRACE-OFF (GRACE-Organic Force Field).

To assess the performance of the GRACE-OFF models, we compare their predictions against established MLIPs, including the MACE-OFF models and, where computationally feasible, the recently introduced Universal Model for Atoms model trained on the Open Molecules 2025 data set (UMA/OMol25), which targets both materials and organic molecular systems.^45^ Due to the comparatively high computational cost of UMA only the small model was used (UMA/OMol25 (S)) in this study. Model performance is assessed using a diverse set of complementary benchmarks. These include single-point energy and force predictions, as well as torsional energy profiles and geometry optimizations. In addition, MD simulations were performed to assess the models’ ability to reproduce condensed phase properties. For five selected organic liquids (methanol, acetone, benzene, *n*-hexane, and *N*-methylacetamide (NMA)), we calculated several thermodynamic properties as well as self-diffusion coefficients. For water, we additionally assessed the temperature-dependent density and radial distribution functions. We also tested whether simulations of biomolecular systems, gas-phase Ala_15_ and solvated crambin, remained stable, without numerical instabilities or unphysical molecular configurations. Given the substantial differences in computational cost across model variants, most of the detailed analysis is restricted to the most promising GRACE-OFF model variants.

Given that our ultimate goal is the application of MLIPs in computationally demanding molecular dynamics simulations, we assess not only predictive accuracy but also practical computational performance. In addition to accuracy benchmarks, we therefore evaluate the computational efficiency of GRACE, MACE, and UMA models by measuring molecular dynamics throughput and memory usage across several GPU architectures. Even highly accurate models may be impractical if excessive memory requirements limit their usability on resource-constrained hardware or if computational cost renders long-time scale simulations infeasible.

The remainder of this manuscript is organized as follows: In Section 2, we provide a short introduction to the GRACE architecture, summarize key characteristics of the training data set, and outline the overall training procedure. Section 3 describes the approaches used for model validation and explains each benchmark in detail. In Section 4, we give an overview of all investigated systems and summarize the simulation settings we used for the MD simulation parts. Section 5 then presents the results obtained for all trained GRACE-OFF models. Finally, in Section 6, we recapitulate our main findings and outline possible directions for future refinements and extensions.

## 2 Model Architecture, Training Data and Training

### 2.1 Model Architecture and Data Set

The GRACE model is a generalization of the linear ACE expansion incorporating semilocal interactions via tree graph basis functions. The basis functions are obtained via efficient recursive evaluation. In contrast to MACE, where ACE features are embedded within a learned message-passing architecture, GRACE constructs the representation directly from recursively evaluated ACE-like tree expansions with minimal architectural design choices. The tree graph basis functions encode geometric correlations over multiple graph levels (tree depths), enabling interactions beyond the local atomic neighborhood. In particular, one-layer models correspond to local ACE-like star graphs, while two-layer models employ two-level tree structures incorporating two-hop neighbor information, extending the effective interaction range to approximately twice the local cutoff radius. In further analogy to ACE, the GRACE energy readout is taken as a nonlinear function of the basis expansion. Further details on the GRACE architecture can be found in ref 41.

We trained the GRACE architecture on the SPICE v2.0 data set (Small-molecule/Protein Interaction Chemical Energies),^26^ which comprises 113,999 molecules represented by a total of 2,008,628 conformations containing 2–110 atoms.[Fn fn1] The data set spans 17 chemical elements and provides reference energies and forces computed at the ωB97M-D3BJ/def2-TZVPPD level of theory. To ensure comparability with the MACE-OFF model family, we followed the protocol in their study^13^ and restricted training to molecules composed of the ten elements H, C, N, O, F, P, S, Cl, Br, and I. To ensure a consistent comparison, all ion pairs (1,426 conformations) were removed and only molecules with a formal net charge of zero were retained. After filtering, the data set consisted of 1,995,993 structures. From this set, we randomly withheld 10,000 structures as a test set and designated an additional 5% of the remaining data as a validation set during training. The training/test and validation sets can be found on zenodo.^46^

### 2.2 Training Details

All GRACE-OFF models were trained for 500 epochs, using the formation energy as the energy target which is defined as the total energy of each conformation minus the sum of the reference energies of the constituent atoms at infinite separation. This quantity retains all position-dependent energy contributions while removing the large, constant offset associated with atomic internal energies, making it the most suitable target for machine-learning-based potential energy models.

For training the GRACE models, following best practices, we employed a learning rate of 0.01 with loss weights of 1 for the energy term and 5 for the force term during the first 300 epochs. After epoch 300, the learning rate was reduced to 0.001, and the loss weights were adjusted to 5 for the energy component and 2 for the force component. Training was performed using the Adam optimizer with a batch size of 32 and a cutoff distance of 6 Å. In total, we trained six GRACE models, three 1-layer models (small, medium, large), referred to as (1L-S/M/L), and three 2-layer models, referred to as (2L-S/M/L) (see Table 1). The total number of learnable parameters and the most important parameters are summarized in Table 1. The full list of parameters to reproduce the training procedure is given in Table S2.

**Table 1:** Hyperparameters of the GRACE Models Used in This Work^a^

# layers	model	# parameters	*l*_max_	max_order	n_rad_max	n_mlp_dens
1	small	58 072	3	3	20	10
1	medium	512 128	4	4	32	12
1	large	1 026 592	4	4	48	16
2	small	235 976	[3, 2]	3	[20, 32]	8
2	medium	901 300	[3, 3]	4	[32, 42]	10
2	large	1 626 848	[4, 3]	4	[32, 48]	12

a *l*_max_ is the maximum per-layer angular-momentum character used for constructing product functions. “max_order” is the maximum product order for the B-functions. “n_rad_max” gives the per-layer maximum number of radial basis functions. “n_mlp_dens” denotes the number of nonlinear readout densities.

To facilitate comparison with the MACE-OFF model family, Table 2 summarizes the total number of trainable parameters and other key parameters of the MACE-OFF23/24 models. The choice of the cutoff radius represents a balance between the size of the local atomic environment required for accurate predictions and the associated computational cost, which increases with the cutoff distance. The chosen value of 6 Å is within the range commonly used for other MLIP architectures (see, e.g., Table 2 for MACE-OFF cutoff radii). As described in “Model architecture and dataset”, the GRACE (2L) models have an interaction radius of up to twice the chosen cutoff. The MACE models achieve a similar effect by using message passing.^40^ Only for the GRACE (1L) models, interactions beyond the cutoff radius are completely ignored.

**Table 2:** Total Number of Trainable Parameters and Other Key Parameters of the MACE-OFF23/24 Models^a^

model	# parameters	cutoff radius [Å]	SPICE version
MACE-OFF23 (S)	694,320	4.5	1
MACE-OFF23 (M)	1,428,368	5.0	1
MACE-OFF24 (M)	1,428,368	6.0	2
MACE-OFF23 (L)	4,707,312	5.0	1

a See also Table 2 in ref 13.

## 3 Validation Strategies

### 3.1 Error Metrics for Energies and Forces

We evaluated the accuracy of the learned potential on *N*_struc_ structures. For each structure *i* ∈ {1, ..., *N*_struc_}, the reference method provides a total energy *E*_*i*_^ref^ and atomic forces **F**_*i,a*_^ref^ for every atom *a* = 1, ..., *N*_*i*_, where *N*_*i*_ is the number of atoms in structure *i*. The model yields corresponding predictions *E*_*i*_^pred^ and **F**_*i,a*_^pred^. We then compare the model predictions to reference values. The error quantities are reported in units of energy per atom (e.g., meV/atom) for energy predictions and units of energy per distance (e.g., meV/Å) for force predictions. Details on the energy and force error metrics are described in the Supporting Information.

### 3.2 Geometry Optimization

While single-point energy and force errors quantify numerical agreement with the reference calculations, geometry optimizations provide a chemically intuitive test of whether an MLIP reproduces the local minima of the underlying QM potential-energy surface. To assess this, geometry optimizations were performed for 100 structures randomly selected from the *GRACE test set*. Starting from identical initial geometries, we optimized the structures using all GRACE-OFF and MACE-OFF model variants discussed here. For reference, we optimized the same set of structures using Psi4 ^47^ at the ωB97M-D3BJ/def2-TZVPPD level of theory, consistent with the energy calculations described for constructing the SPICE data set. We then compare the MLIP- (GRACE-OFF and MACE-OFF) and QM-optimized structures using their root-mean-square deviations (RMSDs) and evaluated the corresponding differences in energy.

### 3.3 Torsion Scans

In many applications, the accurate prediction of minima and maxima on potential energy surfaces as a function of a single rotational degree of freedom is essential. Therefore, a detailed analysis of torsional energy profiles is highly relevant. Let ϕ denote a single torsional (rotational) degree of freedom. For each investigated structure, we compute the MLIP energy prediction

$$E^{\mathrm{pred}}\left(\phi \right),\phi \in \left[0,360^{{}^{\circ}}\right]$$

by rigidly scanning the molecular geometry while varying only the torsional angle ϕ. Each scan thus yields a function (ϕ, *E* ^pred^(ϕ)) over the interval [0, 360°]. We compare this predicted torsional profile to the corresponding DFT scan, denoted *E* ^ref^(ϕ). Three error metrics are evaluated: (1) the mean cumulative area between the two curves ( $\overset{®}{\Delta \mathrm{AUC}}$ ), which provides an overall measure of profile similarity; (2) the mean barrier height (BH) error $\overset{®}{\Delta \mathrm{BH}}$ , which quantifies deviations in the absolute differences between minima and maxima; and (3) the mean absolute barrier height error $\overset{®}{\Delta {\mathrm{BH}}_{\mathrm{abs}}}$ .

These criteria are illustrated in Figure 1, which shows a complete torsion scan for a representative molecule (4,5-Dimethylhex-2-ene) from the TorsionNet data set^48^ obtained with the GRACE-OFF (2L-M) model. The corresponding molecule is also shown, with the scanned torsional angle highlighted in red. The corresponding DFT torsion scan is shown for reference. The orange shaded area between the DFT and the GRACE-OFF (2L-M) curves denotes the ΔAUC error. The two vertical lines denote the barrier heights (BH) for both the DFT and the GRACE-OFF (2L-M) curve. The difference between those two vertical lines corresponds to the ΔBH error. The absolute barrier height error corresponds to ΔBH_abs_ = |ΔBH|. The averaged values ( $\overset{®}{\Delta \mathrm{AUC}}$ , $\overset{®}{\Delta \mathrm{BH}}$ , and $\overset{®}{\Delta {\mathrm{BH}}_{\mathrm{abs}}}$ ) are computed from all individual error values, ΔAUC_*i*_, ΔBH_*i*_, and ΔBH_abs,*i*_, for *i* = 1, ..., *N*_samples_, respectively. Formulas (eqs S1, S2 and S3) and additional details of the computation of all three error metrics are provided in the Supporting Information.

**Figure 1:**
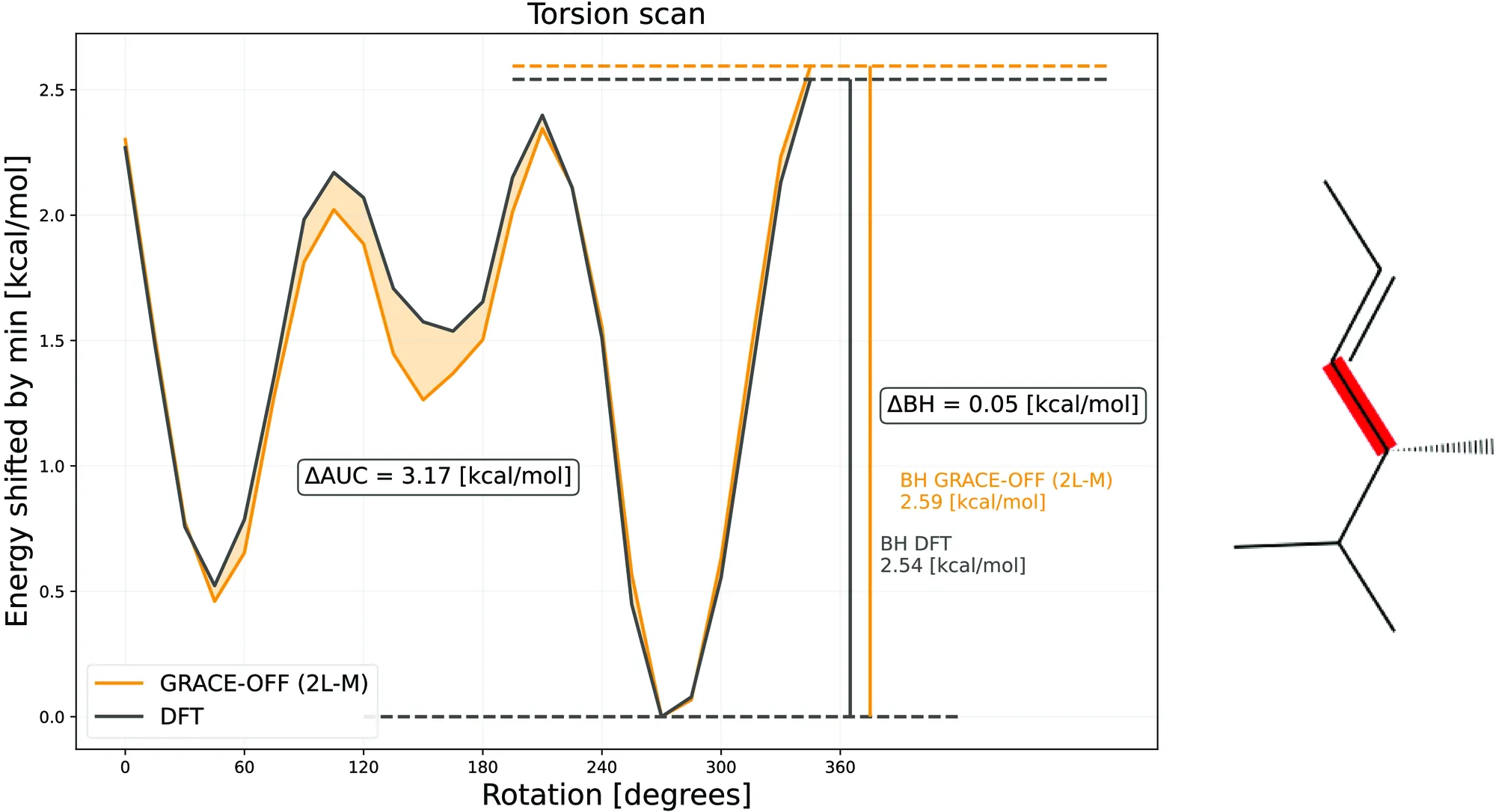
Torsion scan for one single representative molecule (4,5-Dimethylhex-2-ene) computed with the GRACE-OFF (2L-M) model (orange curve) and the corresponding DFT torsion profile (black curve). The shaded orange area between the two curves corresponds to the ΔAUC error. The two vertical lines denote the barrier heights (BH), the difference between the vertical lines corresponds to the ΔBH error. The investigated molecule is shown on the right side, the torsion angle is marked in red.

### 3.4 Condensed Phase Properties

Assessing the accuracy of condensed phase properties is a standard component of classical force-field development.^49–52^ Since trained MLIPs can, in principle, serve as replacements for classical force fields,^53^ such evaluations are equally important for machine-learning potentials. In a recent study,^54^ we examined several ready-to-use transferable MLIPs with an emphasis on their ability to reproduce condensed phase properties. In particular, we evaluated the capability of ANI-2x^55^ and MACE-OFF23 ^13^ to reproduce a range of physicochemical properties for water, methanol, acetone, benzene, and *n*-hexane at room temperature, as well as for *N*-methylacetamide (NMA) at 373.15 K. We also calculated radial distribution functions (RDFs) and diffusion coefficients. Building on this benchmark, we compute the same set of condensed phase properties in the present study for a subset of the GRACE-OFF models (see Section 4).

We report results for the heat of vaporization (Δ*H*_vap_), heat capacity (*C*_*p*_), the coefficient of thermal expansion (α), the isothermal compressibility (κ), diffusion coefficients (*D*), and radial distribution functions (RDFs). Δ*H*_vap_ can be obtained from simulations as

1 $$\Delta H_{vap}=\left\langle E_{gas}\right\rangle -\left\langle E_{liquid}\right\rangle +\mathrm{RT}$$

where *E*_gas_ denotes the energy of a molecule in the gas phase, *E*_liquid_ denotes the energy of one molecule in the liquid phase, *R* denotes the gas constant, and ⟨.⟩ denotes the mean value. κ, *C*_*p*_ and α are computed using the fluctuation formulas given in eqs 2–4:

2 $$\kappa =\frac{1}{k_{B}T}\frac{\langle V^{2}\rangle -\langle V\rangle^{2}}{\left\langle V\right\rangle }=\frac{1}{k_{B}T}\frac{\left\langle \Delta V^{2}\right\rangle }{\left\langle V\right\rangle }$$

3 $$C_{p}=\frac{1}{\mathrm{NR}T^{2}}\left(\left\langle H^{2}\right\rangle -\langle H\rangle^{2}\right)=\frac{1}{\mathrm{NR}T^{2}}\left\langle \Delta H^{2}\right\rangle$$

4 $$\alpha =\frac{\left\langle \mathrm{VH}\right\rangle -\left\langle H\right\rangle \left\langle V\right\rangle }{RT^{2}\left\langle V\right\rangle }=\frac{\mathrm{Cov}\left(\mathrm{VH}\right)}{RT^{2}\left\langle V\right\rangle }$$

where *k*_*B*_ denotes the Boltzmann constant, *N* denotes the number of particles in the simulated system, *T* is the mean temperature and ⟨*Cov*(*VH*)⟩ denotes the covariance of the box volume and the enthalpy. Further details on the derivation of eqs 2–4 can be found, e.g., in Supporting Information of ref 54.

Additionally, we assess the GRACE-OFF models’ ability to reproduce water densities across a range of simulation temperatures. Accordingly, we also present density profiles ρ as a function of temperature *T*, i.e., ρ­(*T*).

### 3.5 Comparison to Other Foundation Models

We compare the GRACE-OFF results to our earlier results obtained with MACE-OFF models. Specifically, we consider the MACE-OFF23 models evaluated in our previous work,^54^ including the small and medium variants, denoted MACE-OFF23 (S) and MACE-OFF23 (M), respectively. In addition, we include results from the recently released MACE-OFF24 (M) model, which was trained on the same SPICE v2.0 data set used for GRACE-OFF. A key difference between the 2023 and 2024 MACE-OFF models is the inclusion of additional water cluster configurations in SPICE v2.0, comprising approximately 1,000 clusters with up to 90 atoms, which are incorporated into the training of MACE-OFF24 (M). To enable a consistent comparison, selected simulations were rerun using this updated MACE-OFF model. At present, no updated small or large MACE-OFF models corresponding to the 2024 release are available. For a subselection of systems (water and *n*-hexane) we also tested the UMA/OMol25 (S) model.^45^

### 3.6 Stability of Biomolecular MD Simulations

In addition to the six liquids, we select the most promising GRACE-OFF model and probe its stability and transferability in MD simulations of well-studied biomolecular systems.^13,31^ Specifically, we run simulations of Ala_15_ in the gas phase and the 46 residue protein crambin in aqueous solution. Both benchmark systems were already used in previous MLIP studies.^13,56^ While the SPICE training data contains structures with up to 110 atoms, Ala_15_ already consists of more than 150 atoms, i.e., it is a larger molecule than any structure seen during training. Unlike single-point energy and force errors, molecular dynamics simulations can reveal whether inaccuracies in the learned potential or extrapolation beyond the training domain result in unstable trajectories or unphysical behavior.^57^ The condensed phase simulations (cf. above) also involve much bigger systems compared to the training data, but their composition is homogeneous. By contrast, the solvated protein forms a heterogeneous environment, and even though crambin is a rather small protein, the system size is considerably larger than those for the simulations of the neat liquids. We therefore use these simulations as a practical stress test for deploying the models in realistic biomolecular simulations, assessing whether the potentials remain stable and produce physically sensible dynamics in regimes beyond the size and complexity of the training configurations.

## 4 Methods

### 4.1 Investigated Systems

To assess the accuracy of the models in predicting energies and forces, the learned potentials were evaluated on 10,000 structures that were not used for training or validation (cf. Section 2.1). We refer to this set of structures as *GRACE test set*. As each entry in the data set provides one energy target and 3N force component target values, where N denotes the number of atoms, this corresponds to 10,000 energy observables and 1,075,074 force components. Since we do not know the split used during the training of the MACE-OFF foundation model, compounds in the *GRACE test set* may well have been part of the MACE-OFF training set. For this reason, we created a second test set consisting of 10,000 randomly chosen structures from the SPICE v2.0 set. This set may contain compounds which either were part of the GRACE-OFF or MACE-OFF training data; therefore we denote it as *biased test set*. The test structures in both the *GRACE test set* and the *biased test set* were randomly selected to minimize bias in the training and evaluation procedure.^46^ For the geometry optimization benchmark, we randomly selected 100 structures from the *GRACE test set*. Dihedral benchmark scans were performed for all 500 molecules in the TorsionNet data set,^48^ which was designed to span a broad range of pharmaceutically relevant chemical space. While the original torsion profiles were generated at the B3LYP/6–31G­(d) level of theory, we instead adopt the torsion scans reported by Kovács et al.^13^ These scans were computed at the ωB97M-D3­(BJ)/def2-TZVPPD level of theory, consistent with the SPICE data set used to train both the MACE-OFF and GRACE-OFF models, therefore ensuring methodological consistency.

To enable a direct comparison, the systems investigated in the condensed phase analysis were the same as in our previous study.^54^ We performed NPT and NVT simulations of six homogeneous liquids: water, methanol, acetone, benzene, *n*-hexane, and *N*-methylacetamide (NMA). Simulations of water, methanol, acetone, benzene, and *n*-hexane were done at room temperature. Since NMA is solid at room temperature and literature data at lower temperatures are not sufficiently accurate,^58^ all major force fields carry out NMA based parametrizations at 373 K.^49,50,59^ Therefore, our NMA simulations were also carried out at 373 K. The simulation systems were constructed using CHARMM-GUI.^60–62^

Biomolecular stability tests were performed for the polyalanine peptide Ala_15_ and the protein crambin. Ala_15_ was simulated in the gas phase and crambin was simulated in an explicit solvent environment. Ala_15_ serves as a minimal, well-defined model system that allows for a clear assessment of intrinsic peptide stability in the absence of solvent effects. Its simple, homogeneous sequence further facilitates the interpretation of structural changes during the simulations. Given its larger size and the presence of charged residues, which are not represented in the training set, crambin serves as a second, more demanding test case. Owing to limited computational resources, the number of solvent molecules was kept intentionally low; however, the system size was sufficient to maintain protein solvation and structural integrity over the course of the simulations. The solvated protein system contains 6438 atoms, making it significantly larger than any molecule/system in the training sets, as well as the systems used in the condensed phase simulations.

### 4.2 Simulation Details

Each of the homogeneous liquids was equilibrated for 10 ns at the force field level (CHARMM General Force Field^63–65^ ) under NPT conditions. All system details, including box sizes, are summarized in Table 3. In addition, single-molecule gas-phase simulations were carried out for each of the six species. These simulations in the gas phase served as an additional stability test and are needed for the computation of the heat of vaporization.

**Table 3:** Simulation Details for Homogeneous Liquids^a^

	water	methanol	acetone	benzene	*n*-hexane	NMA
# molecules	572	323	178	130	98	146
# atoms	1716	1938	1780	1560	1960	1764
*L* [Å]	25.67	27.99	27.95	27.21	27.72	26.97

a *L* denotes the length of the simulation boxes, i.e., *V* = *L* ^3^ after 10 ns force field equilibration under NPT conditions

All MD simulations for the condensed phase analysis of each of the six liquids were performed using the Atomic Simulation Environment (ASE) interface,^66^ using a time step of Δ*t* = 0.5 fs. System properties, such as coordinates, volume, and density, were logged every 100 steps (50 fs).

Initial atomic velocities were drawn from a Maxwell–Boltzmann distribution corresponding to the target temperature. Before the start of dynamics, a short geometry optimization (50 steps) was performed using the BFGS algorithm to relax high-energy steric clashes in the initial configurations. Several simulation protocols were employed:

#### 4.2.1 NVT Ensemble

Performed on bulk liquid systems with periodic boundary conditions to compute self-diffusion coefficients. The center of mass motion was removed to prevent system drift. Temperature was controlled via a Nosé-Hoover Chain thermostat^67^ with a thermostat coupling time of 50 fs. The system was maintained at a temperature of 300 K for all systems except for NMA, which was studied at 373 K. Production runs extended for 2.2 × 10^6^ steps (1.1 ns).

#### 4.2.2 NPT Ensemble

Performed using Isotropic Martyna-Tobias-Klein (MTK) dynamics which provide an integrator, barostat and thermostat.^68^ The system was maintained at a temperature of 300 K—except for NMA—and a pressure of 1.01325 bar for all systems. All NMA simulations were again carried out at 373 K. Coupling constants were set to 50 fs for the thermostat (100 × Δ*t*) and 500 fs for the barostat (1000 × Δ*t*). These simulations were run for 2.2 × 10^6^ steps (1.1 ns).

#### 4.2.3 Gas Phase Dynamics

Performed on single isolated molecules in a nonperiodic 100 Å × 100 Å × 100 Å box under NVT conditions. Temperature was maintained at 373 K for NMA and 300 K for all other systems using a Nosé-Hoover Chain thermostat^67,69^ with a thermostat coupling time of 100 fs. Production runs lasted for 2.2 × 10^6^ steps (1.1 ns).

#### 4.2.4 Density–Temperature Dependency

To test the temperature dependence of liquid water density, we ran several independent simulations at different temperatures and monitored the mean density. Apart from the temperature, all remaining parameters were identical to the described NPT simulation settings. At each temperature *T* ∈ [270, 280, 290, 300, 310, 320, 330] K, we simulated bulk water for 200 ps. The initial 100 ps were discarded as equilibration. The density ρ­(*T*) was calculated as the mean value over the last 100 ps of the simulation data. This analysis was also carried out for the MACE-OFF23­(S) and MACE-OFF24­(M) models. Correspondingly, these MACE simulations were done using ASE.

#### 4.2.5 Gas Phase Dynamics of Ala_15_

The gas-phase simulations of Ala_15_ were performed using Langevin dynamics with a friction coefficient of 0.01 fs^–1^ to maintain an average temperature of 300 K. The integration time step was 1 fs. The total simulation time was 20 ns (2 × 10^7^ steps).

#### 4.2.6 NPT Dynamics of Crambin

NPT molecular dynamics simulations of the protein crambin were performed following the general NPT protocol described above using the isotropic Martyna-Tobias-Klein (MTK) algorithm and an integration step of 1 fs. The starting coordinates were processed using the CHARMM-GUI solvation builder (PDB ID: 1EJG), which created a cubic box with a side length of 41 Å comprising a total of 6,438 atoms. We carried out three independent MD simulations of 500 ps each.

#### 4.2.7 Additional Details on Condensed Phase Analysis

Given the higher computational cost of molecular dynamics analyses and the comparatively lower accuracy exhibited by the 1L models across the single molecule properties benchmarks (see Results), the GRACE-OFF (1L) models are excluded from all condensed phase investigations.

Condensed phase reference values reported in ref 54. were obtained using simulations performed with OpenMM,^70^ whereas all molecular dynamics simulations in the present work were carried out using ASE.^66^ To ensure that this difference in simulation engines does not affect the conclusions, we performed additional test simulations for water using MACE-OFF23­(S) with ASE. The results obtained with ASE and OpenMM, respectively, are in close agreement (see Table S1 in the Supporting Information). In addition, we simplified and shortened the simulation protocol for the condensed phase properties compared to ref 54. First, all NPT/NVT simulations were started from force-field-equilibrated configurations. This choice is motivated by prior observations in ref 54, which indicate rapid equilibration of MACE-based simulations in the condensed phase, suggesting that extended equilibration procedures are not required for the properties considered in this work. Second, in ref 54, we observed that the ANI-2x potential requires substantially longer equilibration times, which motivated three repeats for each NPT simulation to compute condensed phase properties. As this behavior was not observed for either the MACE-OFF or GRACE-OFF models, we performed a single NPT simulation for the investigation of condensed phase properties in this work.

In addition, we performed selected condensed phase simulations using the MACE-OFF24 (M) and the UMA/OMol25 (S) model for water and *n*-hexane. In particular, investigating the MACE-OFF24 (M) model allows us to assess potential improvements relative to the earlier MACE-OFF23 (M) version. These simulations (for both MACE-OFF24­(M) and UMA/OMol25 (S)) were also done using ASE.

## 5 Results

### 5.1 Single Molecule Properties

#### 5.1.1 Energy and Force Prediction

We first evaluated all GRACE-OFF models ((1L) and (2L)) on the *GRACE test set*. The GRACE-OFF (1L) models yield a mean absolute error (MAE) of 1.4–3.6 meV/atom for energies and 32–72 meV/Å for forces. All GRACE-OFF (2L) models exhibit much smaller MAEs of 0.5–1.3 meV/atom for energies and 12–25 meV/Å for forces. For the detailed results, including data for several MACE-OFF models, see Table S3 and Figure S3 in the Supporting Information. The results clearly show that even the small two-layer model (2L-S) already beats the large one-layer model (1L-L). Increasing the model size consistently lowers MAE and RMSE for energies and forces.

MAE and RMSE for energies and forces computed for the *biased test set* for all GRACE-OFF models ((1L) and (2L)), as well as the MACE-OFF23­(S/L) and MACE-OFF24­(M) models are shown in Figure 2. The GRACE-OFF (1L) models exhibit an energy MAE of 1.4–3.5 meV/atom and a force MAE of 32–72 meV/Å. The GRACE-OFF (2L) models exhibit an energy MAE of 0.5–1.2 meV/atom and a force MAE of 12–25 meV/Å. The MACE models show comparable performance with an energy MAE of 0.8–1.3 meV/atom and force MAE of 14–21 meV/Å. The individual numeric values are listed in Table S4 in the Supporting Information. For the GRACE-OFF models, the results are very similar to those for the *GRACE test set*. All two-layer models outperform the one-layer models; the results consistently improve with model size.

**Figure 2:**
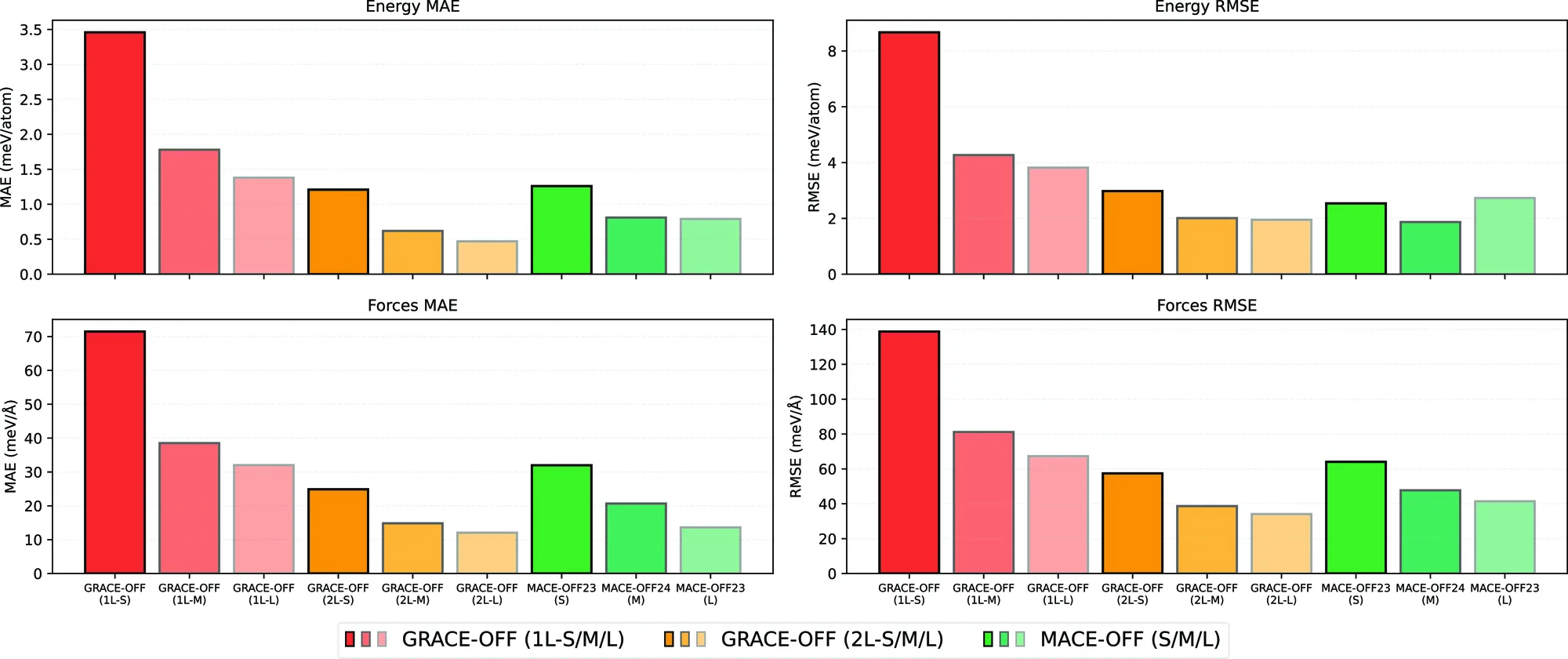
Per-atom MAE and RMSE for energies and forces evaluated on 10,000 random structures from the SPICE data set (*biased test set*). These structures may have been seen by the models during training or validation. GRACE-OFF (1L) models are always depicted in red, GRACE-OFF (2L) models in orange, and the MACE-OFF models in green.

Overall, the performance of the GRACE-OFF (2L) models is comparable to the MACE-OFF23 model family, i.e., GRACE-OFF (2L-S) performs similarly to MACE-OFF23­(S) etc. For both architectures, GRACE and MACE, the accuracy improves most markedly when moving from the small to the medium variants. Overall, GRACE-OFF (2L-L) has the lowest energy and force MAEs among the investigated architectures and model sizes. For the energy MAE and RMSE, the GRACE-OFF (2L) performance consistently improves with increasing model size, even though the energy RMSE of (2L-M) and (2L-S) is very similar.

From the tabulated numbers (Table S4 in the Supporting Information), one sees that for most metrics, the GRACE-OFF (2L) model family performs overall better than the MACE-OFF models. The single exception is energy RMSE for the medium models; here, MACE-OFF24­(M) has a lower value (1.87 meV) than GRACE-OFF (2L-M) (2.01 meV).

#### 5.1.2 Geometry Optimization

Geometry optimizations were performed for all GRACE-OFF (1L-S/M/L and 2L-S/M/L) and MACE-OFF23­(S/L)/MACE-OFF24­(M) models. As shown in Figure S4, the different model variants yield very similar structural accuracy, with mean RMSDs of approximately 0.41 Å and 90% of structural differences remaining below 1 Å. Only ten structures exhibit larger deviations. While the optimized geometries show only minor differences across model architectures (GRACE-OFF vs MACE-OFF) and model sizes (small, medium, and large), the predicted energies are more sensitive to the choice of model. In particular, the mean energy deviation is consistently larger for the GRACE-OFF (1L) models than for the corresponding GRACE-OFF (2L) models and generally decreases with increasing model size.

#### 5.1.3 Torsion Scans

We evaluated the models’ ability to reproduce reference potential energy surfaces obtained from torsion scans of 500 small molecules.^48^ Torsion scans were computed for all GRACE-OFF (1L) and GRACE-OFF (2L) models. For comparison, torsion scans were also computed for the MACE-OFF23­(S/L)/MACE-OFF24­(M) models. We evaluated the metrics ΔAUC, ΔBH, and ΔBH_abs_ for all 500 small molecules, as well as their averaged counterparts $\overset{®}{\Delta \mathrm{AUC}}$ , $\overset{®}{\Delta \mathrm{BH}}$ , and $\overset{®}{\Delta {\mathrm{BH}}_{\mathrm{abs}}}$ (see Section 3). All averaged values ( $\overset{®}{\Delta \mathrm{AUC}}$ , $\overset{®}{\Delta \mathrm{BH}}$ , and $\overset{®}{\Delta {\mathrm{BH}}_{\mathrm{abs}}}$ ) are listed in Table 4 and are given in kcal/mol.

**Table 4:** $\overset{®}{\Delta \mathrm{AUC}}$ Corresponds to the Accumulated Area under the Curve (AUC) Difference between the True DFT and the Model-Predicted Torsion Scan^a^

model	$\overset{®}{\Delta \mathrm{AUC}}$ [kcal/mol]	$\overset{®}{\Delta \mathrm{BH}}$ [kcal/mol]	$\overset{®}{\Delta {\mathrm{BH}}_{\mathrm{abs}}}$ [kcal/mol]
GRACE-OFF (1L-S)	17.31	–0.26	1.10
GRACE-OFF (1L-M)	8.98	–0.11	0.62
GRACE-OFF (1L-L)	7.48	–0.20	0.52
GRACE-OFF (2L-S)	6.04	–0.24	0.44
GRACE-OFF (2L-M)	3.53	–0.13	0.26
GRACE-OFF (2L-L)	**2.64**	–**0.05**	**0.20**
MACE-OFF23 (S)	7.75	–0.12	0.54
MACE-OFF24 (M)	5.72	–0.21	0.41
MACE-OFF23 (L)	3.36	–0.11	0.24

a $\overset{®}{\Delta \mathrm{BH}}$ corresponds to the mean over all barrier height (BH) errors. Negative errors can cancel out positive errors, and vice versa. That is, $\overset{®}{\Delta \mathrm{BH}}<0$ means that the model underestimates the barrier height on average, $\overset{®}{\Delta \mathrm{BH}}>0$ means that the model overestimates the barrier height on average. $\overset{®}{\Delta {\mathrm{BH}}_{\mathrm{abs}}}$ corresponds to the mean over the absolute BH errors.

In correspondence to energy and force predictions, the GRACE-OFF (2L) models exhibit overall better performance compared to the GRACE-OFF (1L) models. In addition, increasing model size (S/M/L) leads to improved predictive accuracy. All 2L models outperform all 1L models, with the GRACE-OFF (2L-L) model yielding the smallest averaged errors across all investigated torsion-profile validation metrics. Interestingly, all models—including the MACE-OFF model family—systematically underestimate the total barrier height, i.e., $\overset{®}{\Delta \mathrm{BH}}<0$ (see Table 4).

The GRACE-OFF (1L) models show the largest prediction errors. Nevertheless, even these comparatively simple models appear to capture the correct positions of local minima and maxima along the torsion profiles. On average, the GRACE-OFF (2L) models provide the most accurate predictions. A comparison across model sizes further indicates that the GRACE-OFF models consistently outperform the MACE-OFF models. In particular, the GRACE-OFF (2L-M) model even reproduces the DFT reference data approximately as good as the MACE-OFF23 (L) model.

### 5.2 Condensed Phase Analysis

We first present physicochemical/thermodynamic properties of water, methanol, acetone, benzene, and *n*-hexane at 300 K, and *N*-methylacetamide (NMA) at 373 K obtained using all GRACE-OFF (2L) models and compare them to the results obtained with the small and medium MACE-OFF23 models as reported in ref 54 (Section 5.2.1). Since some MACE-OFF23­(M) results were in poor agreement with experiment and since it has been superseded by MACE-OFF24­(M), we report results for water and hexane obtained with MACE-OFF24, as well as UMA/OMol25 (S)^45^ (Section 5.2.2). Finally, we report additional water properties, in particular the density over a range of temperatures in Section 5.2.3.

#### 5.2.1 Physicochemical/Thermodynamic Properties of Selected Liquids

We start our analysis with a comparison of predicted densities. The deviation Δρ = ρ^pred^ – ρ^exp^ for all GRACE-OFF (2L) models and for the MACE-OFF23 (S/M) models is shown in Figure 3. Overall, both model families tend to overestimate densities, with notable exceptions for the MACE-OFF23 (M) model, which severely underestimates the densities of acetone and hexane, and to a lesser degree that of NMA. The deviation of all GRACE-OFF models from the experimental density of water is ≤ 0.01 g/mL, while MACE-OFF23­(S) deviates by 0.12 g/mL and MACE-OFF23­(M) by 0.18 g/mL (see Table S5 in the Supporting Information). Overall, the predicted densities using GRACE-OFF are better and more consistent than those using MACE-OFF23. In particular, the performance of MACE-OFF23­(M) is erratic; e.g., while it almost perfectly predicts the density of benzene, the density of water is massively overestimated and that of hexane massively underestimated.

**Figure 3:**
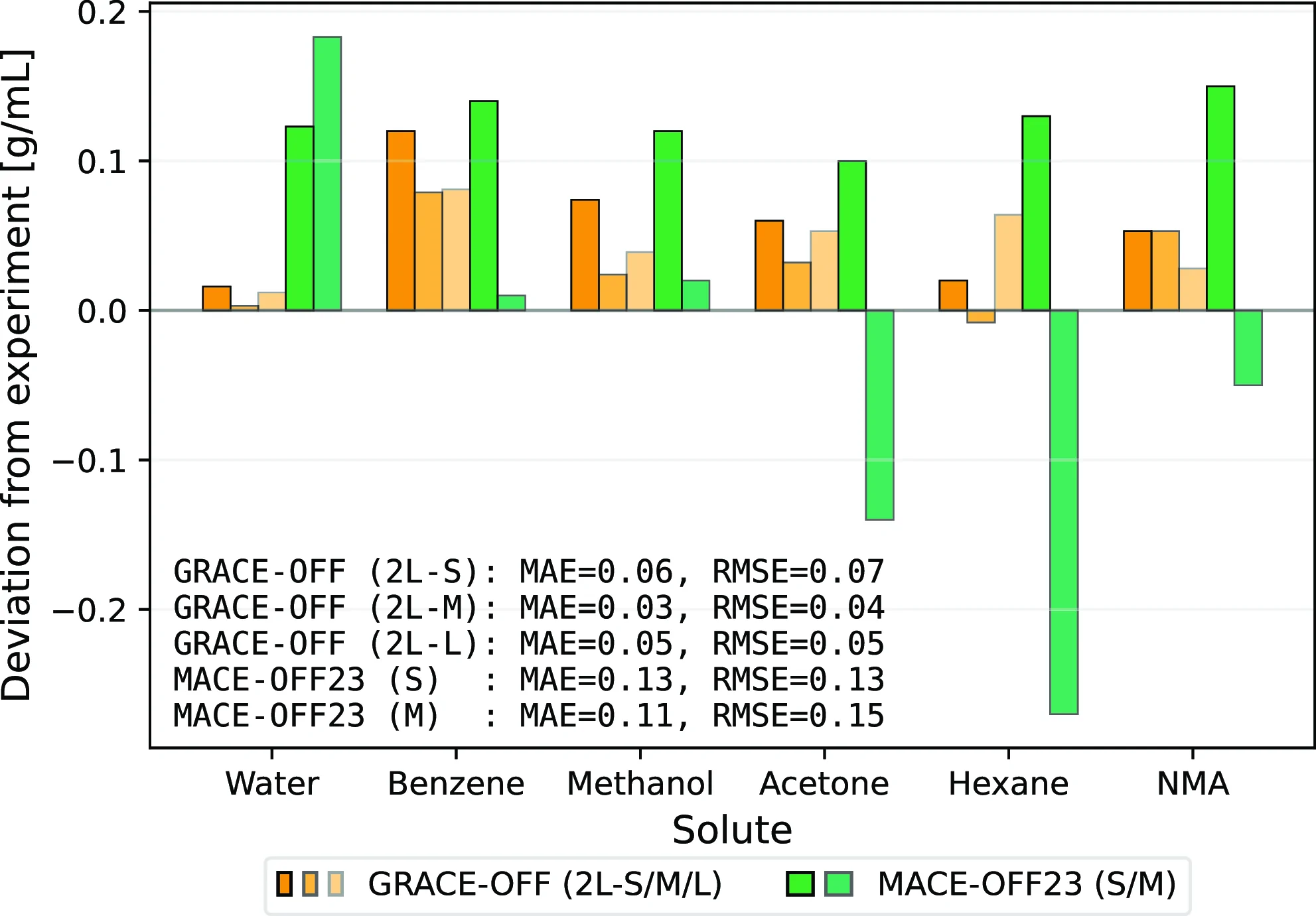
Density deviation from experimental values for all six investigated liquids for the GRACE-OFF (2L) models and MACE-OFF23­(S/M) models (all reported results for the MACE-OFF models are taken from ref 54).

To capture the overall performance for all six liquids, we report the MAE_ρ_,

5 $${\mathrm{MAE}}_{\rho }=\frac{1}{N_{\mathrm{liquids}}}\sum_{i=1}^{N_{\mathrm{liquids}}}\left|\rho_{i}^{\mathrm{pred}}-\rho_{i}^{\exp}\right|$$

where ρ_*i*_^pred^ denotes the predicted density for liquid *i* ∈ {1, ..., *N*_liquids_}, and ρ_*i*_^exp^ denotes the respective experimental density. The numerical results are listed in Table 5. The density MAE_ρ_ for the GRACE-OFF (2L) models ranges from 0.03–0.06 g/mL, with the (2L-M) model performing the best. The MAE_ρ_ for both MACE-OFF models is significantly larger, 0.13 for the small and 0.11 g/mL for the medium model.

**Table 5:** Mean Absolute Error (MAE) Over All Six Liquids (Water, Benzene, Methanol, Acetone, Hexane and NMA) of the Thermodynamic Properties Δ*H*_vap_ (Heat of Vaporization), *C*_*p*_ (Heat Capacity), κ (Isothermal Compressibility), α (Coefficient of Thermal Expansion), and ρ (Density)^a^

	Δ*H*_vap_ [kJ/mol]	*C*_*p*_ [cal/(g K)]	κ [10^–4^/bar]	α [10^–2^/K]	ρ [g/mL]
GRACE-OFF (2L-S)	4.64	0.77	0.48	0.03	0.06
GRACE-OFF (2L-M)	5.23	0.73	**0.28**	**0.02**	**0.03**
GRACE-OFF (2L-L)	**2.48**	**0.72**	0.33	**0.02**	0.05
MACE-OFF23 (S)^b^	2.84	0.93	0.78	0.12	0.13
MACE-OFF23 (M)^b^	8.04	0.89	71.31	0.90	0.11

a The lowest MAE value in each column is highlighted in bold.

b Values from ref 54.

Next, we turn to all other properties computed from NPT simulations, i.e., the heat of vaporization (Δ*H*_vap_), heat capacity (*C*_*p*_), isothermal compressibility (κ), and thermal expansion coefficient (α). Corresponding to eq 5, we computed MAE_γ_ for all investigated properties γ ∈ {Δ*H*_vap_, *C*_*p*_, κ, α}. Table 5 summarizes the MAE_γ_ over all six liquids for each of these properties. The MACE-OFF23­(S/M) MAEs are computed from the results reported in ref 54. Across the full property set, all three GRACE-OFF models reproduce the experimental values reasonably well. With the exception of Δ*H*_vap_, even the small model (2L-S) has lower MAEs than either of the MACE-OFF23 models. GRACE-OFF (2L-M) achieves the best overall performance for κ, α, and ρ (MAE of 0.28 [10^–4^/bar], 0.02 [10^–2^/K] and 0.03 [g/mL], respectively). GRACE-OFF (2L-L) provides the most accurate prediction for Δ*H*_vap_ (MAE 2.48 [kJ/mol]) and *C*_*p*_ (MAE 0.72 [cal/(g,K)]). Both MACE-OFF23 models show substantially larger deviations for several properties, most prominently for κ and α. The individual values for each liquid and each property are summarized in the Supporting Information in Tables S5–S10.

To investigate the convergence of the simulated systems, we report Δ*H*_vap_, *C*_*p*_, α, and κ as functions of increasing simulation time in Figure S1 in the Supporting Information for the GRACE-OFF models. Convergence of the MACE models was demonstrated previously (see Figure 5a in ref 54). The 1.1 ns simulation trajectory was divided into ten windows, each containing an additional 100 ps compared to the previous window, i.e., the first data point was calculated from the first 100 ps of the production trajectory, whereas the final data point was obtained from the full 1 ns production trajectory. The convergence analysis suggests that benzene might benefit from longer simulation times. We, therefore, performed an additional independent 2.2 ns simulation of benzene. The first 200 ps were discarded as equilibration, and all thermodynamic properties were evaluated over disjoint 250 ps windows from the remaining 2 ns. The results indicate that the properties reach stable values after approximately 500 ps (see Figure S2). Based on this analysis, we also computed all thermodynamic properties for benzene from the final nanosecond of this additional simulation. The results from both, the final nanosecond of the original 1.1 ns simulation and the final nanosecond of the additional 2.2 ns simulation, are reported in Table S6. While the results differ substantially between the different models (GRACE-OFF (2L) and MACE) and model sizes (S/M/L), the values obtained from the two simulations remain overall in good agreement. For example, the heat of vaporization varies by almost 10 kJ/mol across the different models, ranging from 27.86 kJ/mol for the (2L-S) model to 37.06 kJ/mol for the (2L-M) model. In contrast, the largest difference between the two simulations for a given model is less than 2.8 kJ/mol, observed for the (2L-L) model, which yields values of 31.18 and 33.88 kJ/mol for the original and additional simulation, respectively. Similarly, κ varies by approximately 0.2 × 10^–4^/bar across the different models, from 0.49 × 10^–4^/bar for the (2L-L) model to 0.70 × 10^–4^/bar for the (2L-M) model. The largest difference between the two simulations is less than 0.12 × 10^–4^/bar, observed for the (2L-S) model, with values of 0.52 and 0.63 × 10^–4^/bar for the original and additional simulation, respectively.

Finally, for GRACE-OFF (2L-S) and (2L-M) we also computed the self-diffusion coefficients (D) for water, acetone, methanol, n–hexane and benzene and compare them to the experimental values;^71^ see Figure 4. The numeric values of D for each model and liquid are listed in Table S11. NMA was excluded from this analysis as no experimental value was available at 373 K. We did not compute D for GRACE-OFF (2L-L) since the large model is computationally more costly and did not lead to systematic improvements for the thermodynamic properties just presented. Similarly, in ref 54. diffusion constants were only computed for MACE-OFF23­(S). As one sees in Figure 4, diffusion constants obtained with either GRACE-OFF model have the correct order of magnitude and show overall low deviations from the experimental values (MAE 0.39·10^–9^ m^2^/s for GRACE-OFF (2L-S) and 0.42 × 10^–9^ m^2^/s for GRACE-OFF (2L-M)). In contrast, diffusion constants obtained from the MACE-OFF23­(S) model display markedly less consistent behavior, including severe outliers (most prominently for methanol) and strong underestimation for the apolar liquids. Only the diffusion coefficients for water and acetone are in good agreement with experiment.

**Figure 4:**
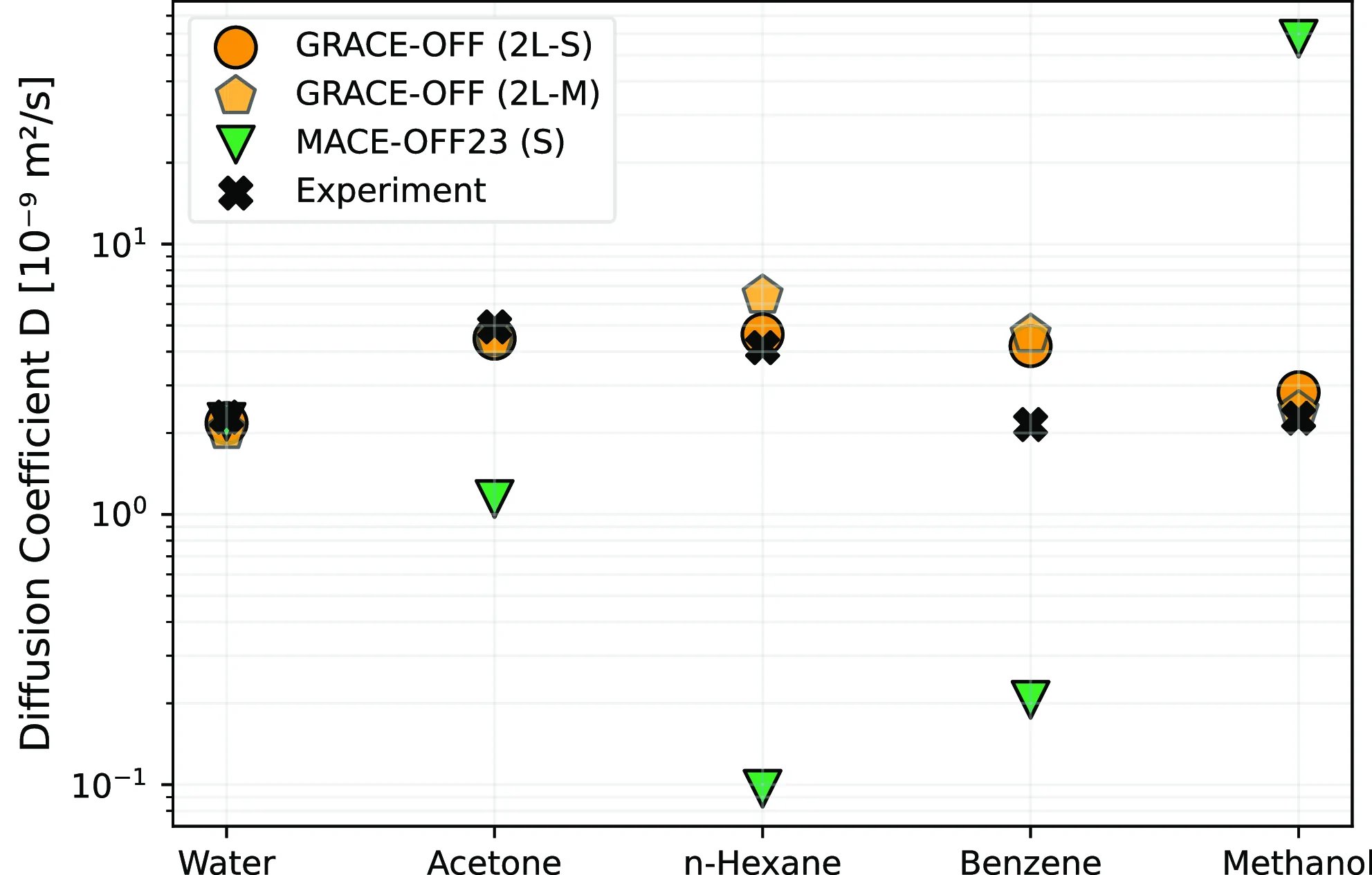
Self-diffusion coefficients for water, acetone, *n*-hexane, benzene and methanol, for the GRACE-OFF (2L-S/M) and MACE-OFF23­(S) models. The black crosses denote experimental values taken from ref 71. MACE-OFF (S) diffusion coefficients were taken as reported in ref 54.

#### 5.2.2 Additional Simulations with MACE-OFF24­(M) and UMA/OMol25 (S) for Water and Hexane

For water and hexane, we performed additional NPT simulations using the MACE-OFF24­(M) and the UMA/OMol25 (S) model.^45^ Figure 5 summarizes the thermodynamic properties for water obtained with all models considered, i.e., the GRACE-OFF (2L) models and the MACE-OFF23­(S/M) models (see also Section 5.2.1), as well as MACE-OFF24 and UMA/OMol25 (S).

**Figure 5:**
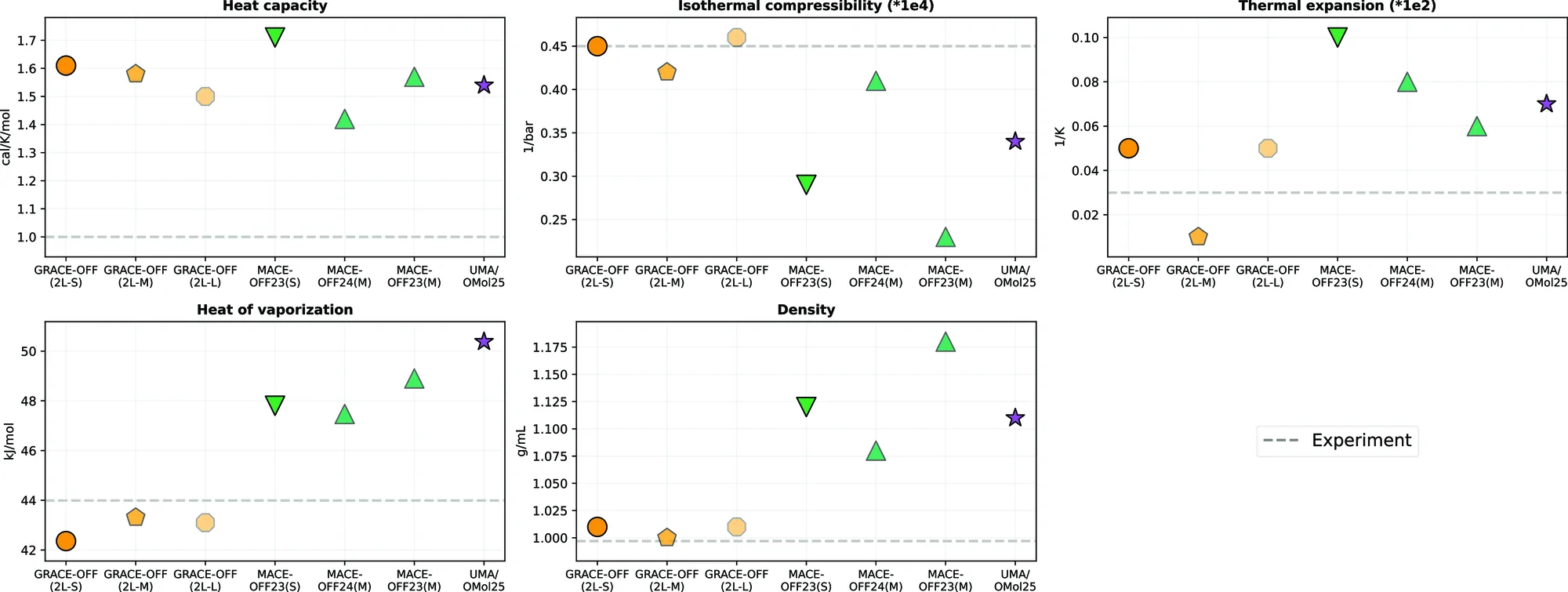
Thermodynamic properties of bulk water are shown for GRACE-OFF (2L), circle shaped markers), MACE-OFF (MACE-OFF23 (S/M), MACE-OFF24 (M), triangle shaped markers), and UMA/OMol25 (S, stars). Results for MACE-OFF23 (S/M) are taken from ref 54. Colors denote model family. Dashed horizontal lines indicate experimental reference values.^72^

The heat capacity is noticeably overestimated across all models. The isothermal compressibility is best reproduced by the three GRACE-OFF models, and the same trend is observed for the heat of vaporization and the density. The UMA/OMol25 (S) model reproduces the physical properties reasonably well but does not reach the accuracy achieved by the GRACE-OFF models. Comparing MACE-OFF23 (M) and MACE-OFF24 (M), the newer model shows improved accuracy, particularly for the density and the isothermal compressibility. The tabulated values for bulk water are provided in Table S5.

The MACE-OFF24 (M) results for *n*-hexane are a clear improvement compared to MACE-OFF23 (M) results (see Table S9). However, the GRACE-OFF models overall are still in better agreement with experiment. For hexane, UMA/OMol25 (S) mostly performs well, in particular the heat of vaporization is well reproduced. However, the density of hexane is best reproduced by GRACE-OFF (2L-M), whereas UMA/OMol25 (S) overestimates ρ by more than 10%.

#### 5.2.3 Additional Water Properties

Given the unique properties of water and its central role as a solvent, we investigated several additional properties of bulk water. Figure 6 shows the water RDFs (O–O, H–O, and H–H) for the three GRACE-OFF (2L) models computed from 1 ns NPT simulations. The corresponding experimental RDF curves are also included.^73^ For all three RDFs, the GRACE-OFF models match the experimental RDF curves very well, with GRACE-OFF (2L-M) showing the best agreement with the experimental data. However, some deviations remain: the first O–O peak is overestimated, the second O–O peak is insufficiently resolved, and the O–H peaks are too pronounced. For the (2L-S) and (2L-L) models, the positions of the first minimum and maximum are also slightly shifted to larger distances.

**Figure 6:**
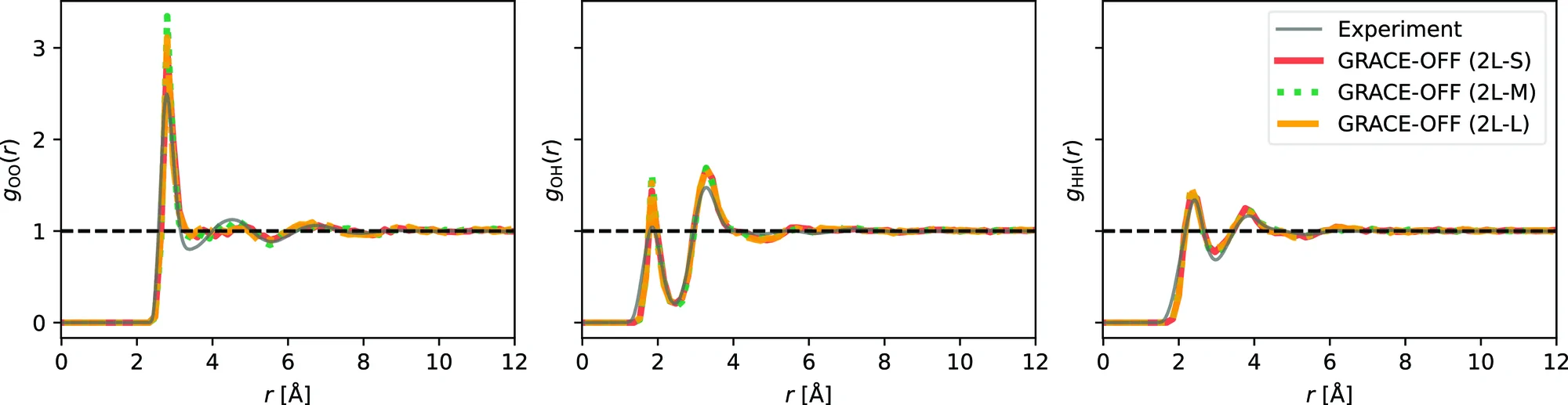
Radial distribution functions for the three GRACE-OFF (2L) models together with the experimental values are taken from ref 73. From left to right: the oxygen–oxygen, oxygen–hydrogen and hydrogen–hydrogen RDF is shown.

Figure 7 reports the density of liquid water as a function of temperature, i.e., ρ­(*T*), for *T* ∈ [270, 280, 290, 300, 310, 320, 330]. All numerical values are listed in Table S12. Overall, the densities predicted by the three GRACE-OFF (2L) models are in very good agreement with experimental reference data. All models correctly capture the expected decrease in density with increasing temperature. The GRACE-OFF (2L-S/L) models slightly overestimate the density, whereas the GRACE-OFF (2L-M) model reproduces the experimental density curve almost perfectly. In contrast, the MACE-OFF models systematically overestimate the density by approximately 5–15%. Notably, the comparison includes the most recent MACE-OFF24­(M) model, which was trained on the same data set as the GRACE-OFF models. Not only is the density of the MACE-OFF models significantly too high, but the decrease of the density as a function of temperature is too steep. Very recently, a MACE-OFF variant augmented by long-range corrections, called MACELES-OFF (MACE Latent Ewald Summation),^74^ was released. In comparison to the data shown there (see Figure 7c of ref 74), all GRACE-OFF (2L) models also outperform MACELES-OFF.

**Figure 7:**
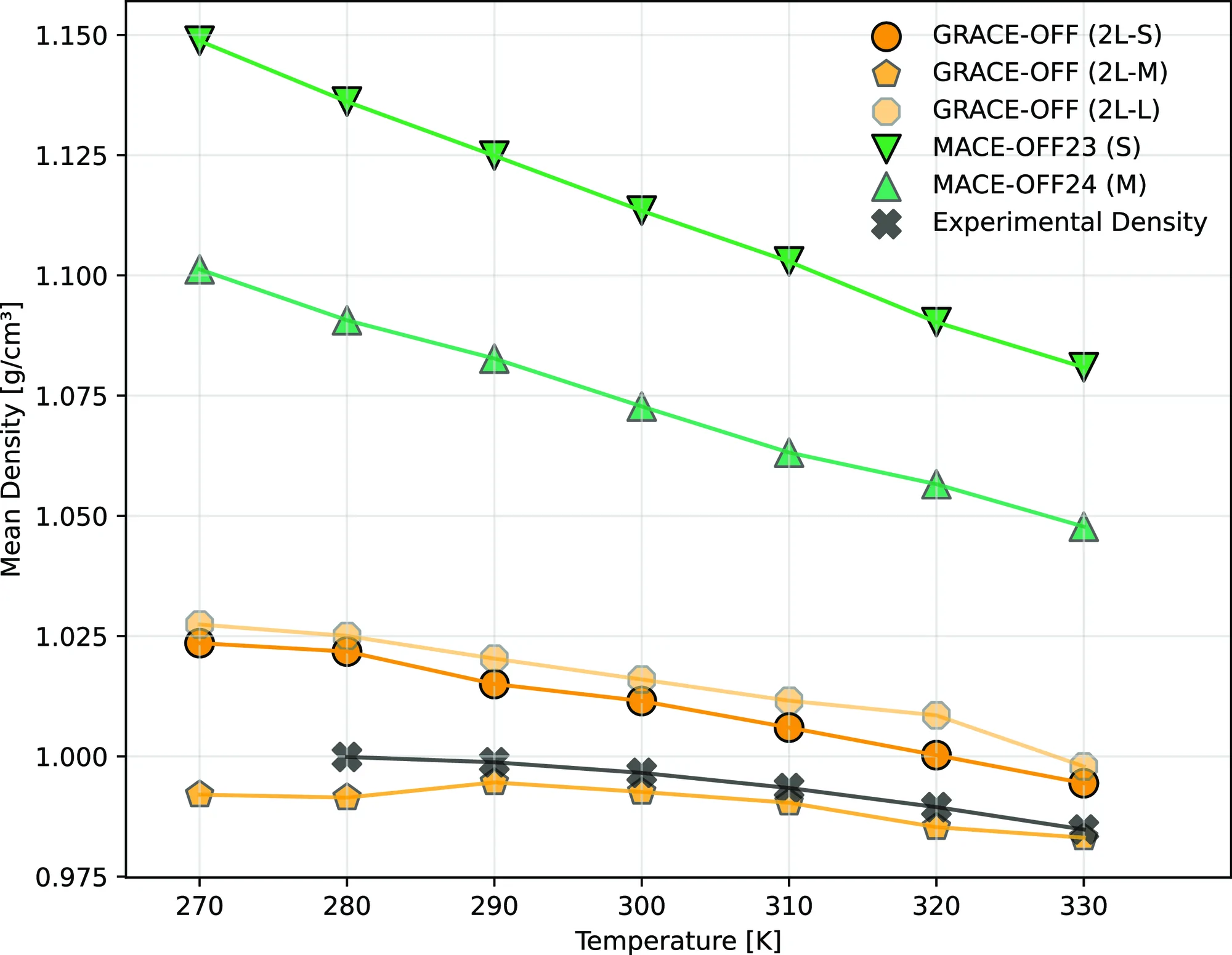
Density of bulk water as a function of temperature for GRACE-OFF (2L) and MACE-OFF23­(S) and MACE-OFF24­(M) models. Experimental values were taken from ref 72 (p. 986 and p. 991).

### 5.3 Biomolecular Simulations

In this section, we briefly summarize the results of our biomolecular stability tests. All biomolecular simulations were done with the GRACE-OFF (2L-M) model. As a first benchmark, we report results for gas-phase MD simulations of the polypeptide Ala_15_. All simulation runs for Ala_15_ remained stable, meaning that no unphysical expansion or collapse of the system within the simulation box and no bond breaking were observed. The observed folding behavior aligned well with previous computational studies of polyalanine peptides.^13,56^ Such folding behavior has also been observed experimentally.^75^ Figure 8 shows the fraction of residues adopting different secondary-structure motifs as a function of time, including α-helical and 3_10_-helical conformations, as well as turn, bend, coil, and loop structures. For comparison, see also Figure 9 in ref 13.

**Figure 8:**
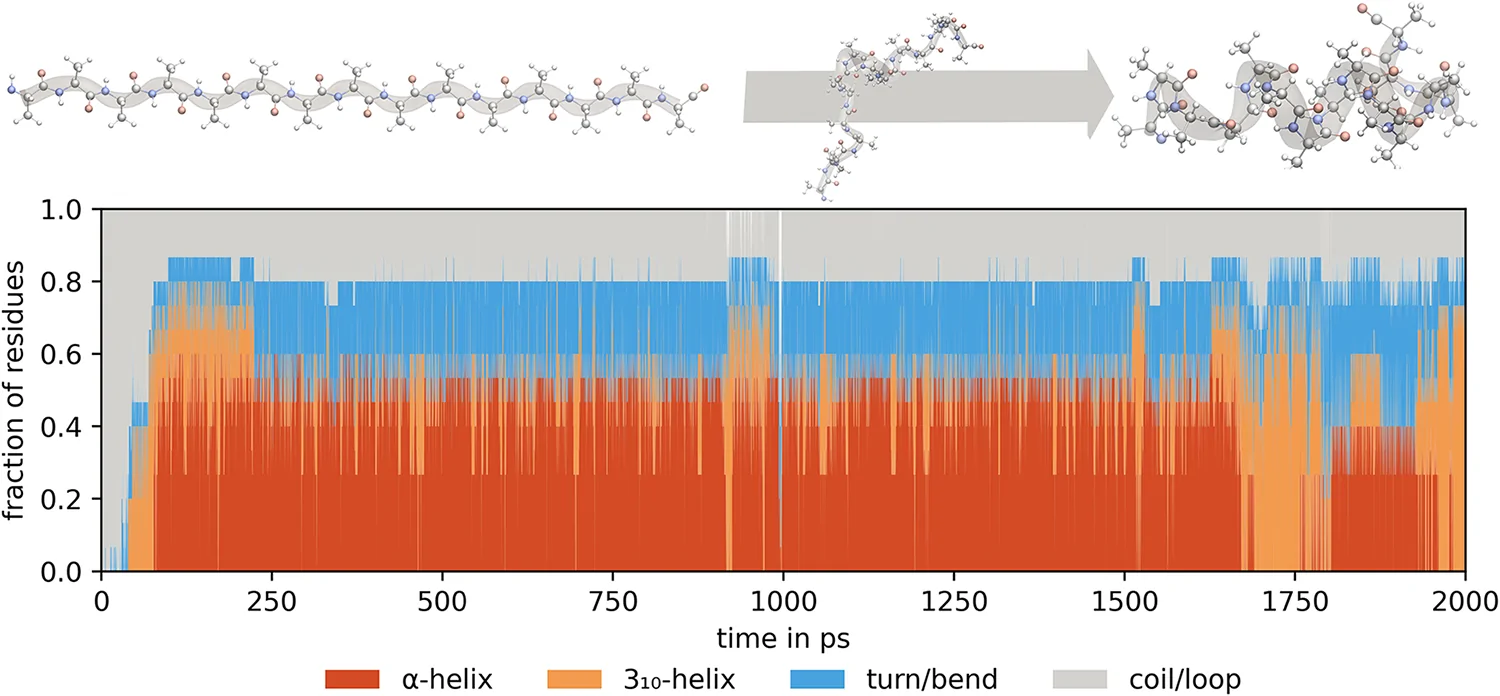
Conformational evolution of the polyalanine peptide Ala_15_. Starting from an extended coil conformation, the peptide rapidly folds into an α-helical structure within the first few picoseconds. Throughout the remainder of the simulation, the peptide predominantly remains α-helical, with occasional transitions to a 3_10_ helix.

**Figure 9:**
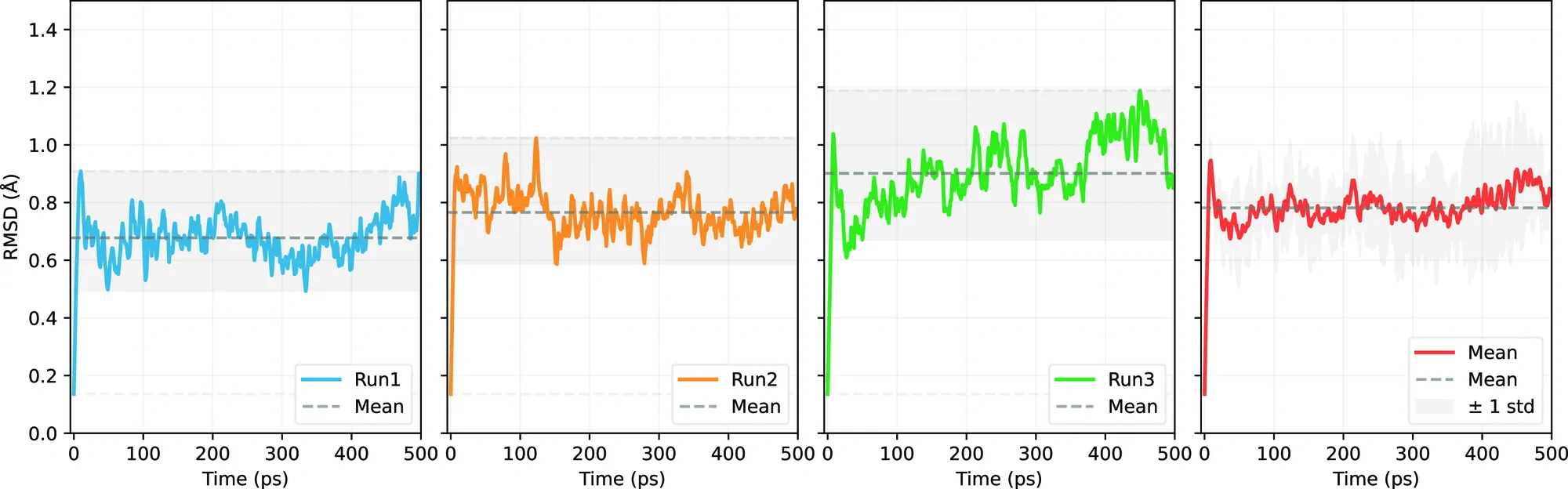
Root-mean-square deviation (RMSD) of crambin relative to the initial structure as a function of simulation time. The first three plots (from left to right) show the RMSD for all three individual simulation runs. In the fourth plot (on the right side), the solid red line shows the mean RMSD averaged over three independent simulations, while the shaded gray region indicates the corresponding standard deviation over the three independent runs. The dashed horizontal line denotes the mean in all four subplots.

Second, we report results from MD simulations of the small protein crambin solvated in explicit water. Structural stability is assessed by monitoring the root-mean-square deviation (RMSD) of all protein atoms relative to the initial structure. Figure 9 presents the RMSD as a function of simulation time. The first three subplots (from left to right) show the individual trajectories; in each, the shaded gray area denotes the range between the minimum and maximum RMSD values after the initial relaxation phase. The fourth panel displays the mean RMSD averaged over three independent simulations (solid red line), with the shaded gray region indicating one standard deviation around the mean. The first and second runs exhibit slightly smaller structural deviations and, consequently, reduced RMSD fluctuations compared to the third run. Overall, however, after an initial relaxation phase, the RMSD in all three simulations fluctuates between approximately 0.5 and 1.2 Å.

### 5.4 Computational Performance

To assess computational performance, we simulated a cubic water box with a side length of 24.8 Å containing 572 water molecules (1716 atoms). Performance tests were carried out on five NVIDIA GPUs with varying memory capacities and computational speeds. Figure 10 presents a direct throughput comparison (ns/day) of the GRACE-OFF, MACE-OFF, and UMA/OMol25 (S) models on two representative GPUs (NVIDIA GeForce RTX 4090 and NVIDIA A100) in both single precision (float32) and double precision (float64). Results for the remaining GPUs are provided in the Supporting Information in Figure S5. Across all tested hardware, the GRACE-OFF models consistently achieve high simulation speeds. This observation aligns with the original GRACE study;^76^ however, unlike that work, we did not employ LAMMPS to further accelerate the simulations. Both GRACE-OFF (2L-M) and GRACE-OFF (2L-L) are substantially faster than the medium and large MACE variants, respectively, in both single- and double-precision calculations. The MACE large model could not be executed on the RTX 4090 due to an out-of-memory error. The same limitation was encountered for double-precision calculations on the RTX 6000 Ada as well as on the A40 and L40S GPUs. In contrast, all GRACE-OFF model variants ran successfully on all tested GPUs in both single and double precision, indicating substantially lower memory requirements.

**Figure 10:**
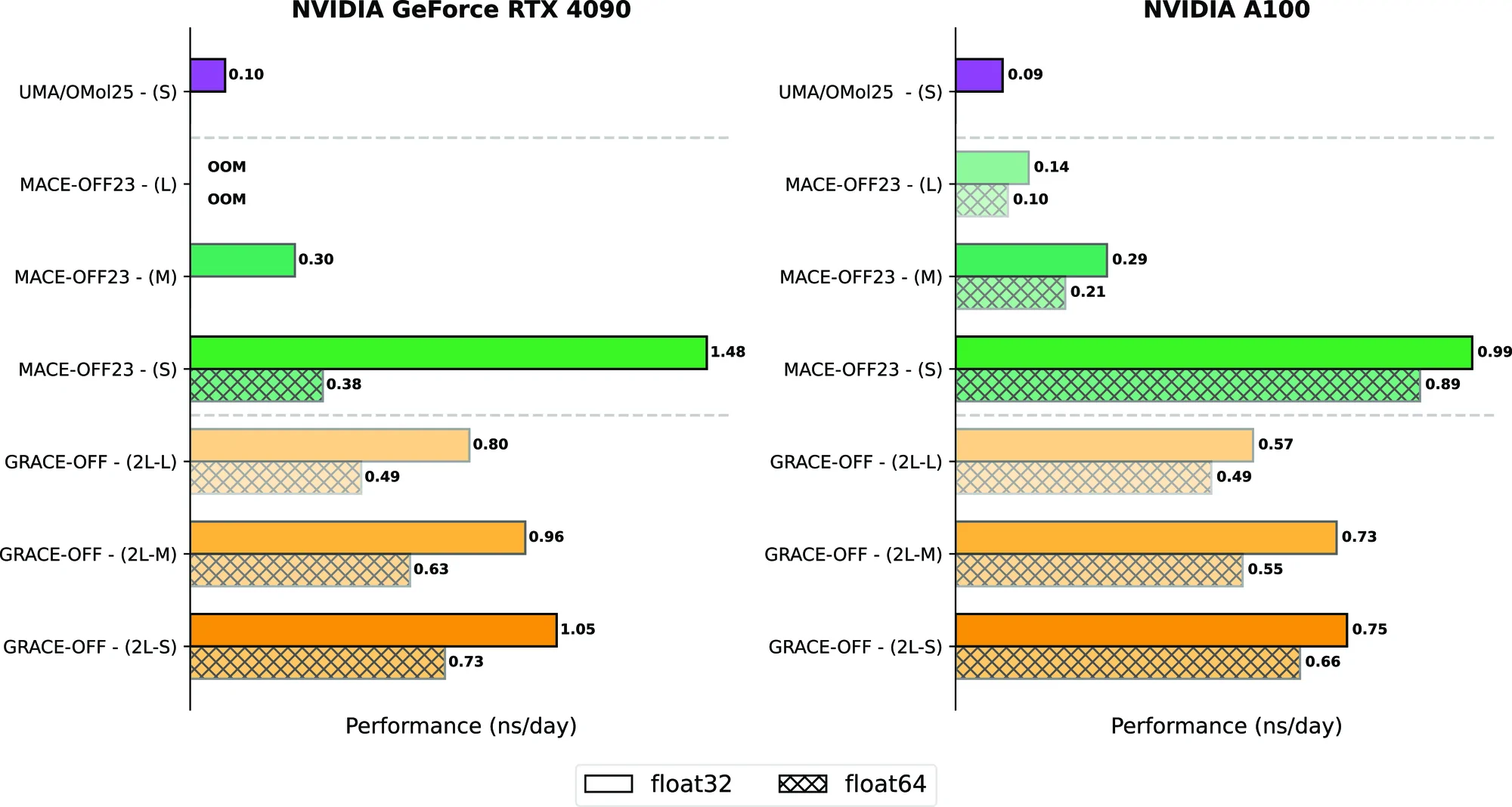
Computational performance in ns/day for different GRACE-OFF and MACE-OFF models as well as for UMA/OMol25 (S) across different NVIDIA GPUs. Results are shown for single-precision (float32) and double-precision (float64) simulations. We show results for consumer-grade hardware (NVIDIA GeForce RTX 4090) on the left as well as for a data-center-class GPU (NVIDIA A100) on the right side. If a model could not be run, this is indicated by the OOM (out of memory) label next to the model’s name. Additional results for the performance on a NVIDIA A40, NVIDIA L40S and NVIDIA RTX 6000 Ada are shown in Figure S5 in the Supporting Information.

The UMA/OMol25 (S) model is the slowest model considered in this benchmark analysis, performing below GRACE-OFF and MACE-OFF small and medium models, exhibiting throughput comparable to the MACE-OFF (L) model. However, it should be noted that, although the computational cost of the UMA/OMol25 (S) model is too high to enable meaningful simulations on standard cluster setups, it does not produce an out-of-memory error. This contrasts with the MACE-OFF23­(L) model, which cannot be executed on consumer-grade GPUs such as the NVIDIA RTX 4090 due to memory limitations.

Overall, these benchmarks demonstrate that GRACE-OFF delivers the most consistent and robust performance across both data-center (A100) and consumer-grade (RTX 4090) GPUs, with particularly strong advantages for the medium and large model variants.

## 6 Summary and Outlook

In this work, we demonstrated that the GRACE neural network architecture can be successfully trained on the SPICE v2.0 data set for the prediction of potential energy surfaces and seamlessly integrated into an established molecular dynamics engine to perform stable and accurate molecular dynamics simulations. By training and evaluating multiple variants of the GRACE architecture, we systematically assessed the impact of network depth and model size on predictive accuracy across a diverse set of validation tasks.

Our results show that increasing model complexity, both in terms of network depth and size, consistently improves performance. In particular, each GRACE-OFF (2L) model outperforms all 1L counterparts across torsion-profile validation metrics as well as energy and force predictions. The GRACE-OFF (2L-L) model yielded the smallest averaged errors for these single-molecule properties.

Beyond gas-phase benchmarks, the GRACE-OFF models demonstrate strong transferability to condensed phase simulations. While some deviations from experimental reference data remain, the GRACE-OFF models generally provide good agreement across the investigated properties. In particular, the GRACE-OFF (2L) variants provide good agreement for thermodynamic, structural, and dynamic properties of a range of organic liquids as well as bulk water, achieving consistently closer agreement with experimental reference data compared to the MACE-OFF models. Properties obtained from both NPT simulations (e.g., densities and heats of vaporization) and NVT simulations (e.g., self-diffusion coefficients) are generally well reproduced across all investigated systems, indicating that the GRACE architecture captures relevant many-body and dynamical effects despite being trained exclusively on single-point energies and mostly gas-phase reference data.

Liquid water represents a particularly stringent test case. All GRACE-OFF (2L) models accurately reproduce key properties of liquid water, including heat of vaporization and density, as well as radial distribution functions. While MLIPs reproduce the expected decrease in density with increasing temperature, all GRACE-OFF (2L) models predict densities in very close agreement with experiment, substantially reducing the systematic overestimation observed for the MACE-OFF models. Notably, the GRACE-OFF (2L-M) model reproduces experimental density values with high accuracy across the investigated temperature range. Notably, the GRACE-OFF (2L) models also outperform long-range-enhanced MACE-OFF variants in reproducing properties of condensed-phase water simulations,^74^ with the latter still showing less accurate density predictions compared to the GRACE-OFF models.

Across all benchmarks, the GRACE-OFF (2L-M) model offers the most favorable balance between predictive accuracy and computational efficiency. It is approximately an order of magnitude faster than the UMA/OMol25 (S) model and around three times faster than the MACE-OFF (M) variant, while maintaining a high degree of accuracy. In addition to higher throughput, the GRACE-OFF models exhibit significantly lower memory requirements. For example, molecular dynamics simulations of a 572-molecule water box could not be executed with the MACE-OFF23 (L) model due to memory limitations, whereas all GRACE-OFF variants ran without such constraints. This is particularly notable when comparing various model sizes, given that the total number of trainable parameters in the MACE models exceeds that of the GRACE-OFF variants (see Tables 1 and 2); this suggests that despite having fewer parameters, the GRACE architecture can capture meaningful behavior efficiently.

Overall, the GRACE-OFF model family provides a new and versatile set of foundation models suitable for a wide range of applications. The models are capable of delivering accurate predictions for both molecular conformational energetics and condensed phase properties. The demonstrated balance between accuracy, transferability, and computational feasibility highlights the models’ potential as a general-purpose machine-learned force field family for molecular simulations. While this study validates the GRACE architecture across a range of benchmarks, further improvements could be achieved through systematic exploration of architectural choices, hyperparameter optimization, and training on diverse data sets, paving the way for tailored applications in specific molecular domains.

## Supplementary Material



## Data Availability

All scripts used to compute the results presented here, as well as the input PDB files and all GRACE-OFF models, can be found in the GitHub repository https://github.com/heid-lab/grace-off/. The data used for training the GRACE-OFF models, as well as the corresponding evaluation data sets, are available at DOI: https://doi.org/10.5281/zenodo.18662354. We provide the following files: SPICE_V2.0_cleaned.xyz: contains all structures with their corresponding energies and forces, extracted from the original SPICE v2.0 repository. This data set forms the basis for the training, validation, and testing splits used in this work. input.yaml: example input script for fitting the 2-layer medium GRACE-OFF model. training_set.pkl.gz and test_set.pkl.gz: contain the training and validation splits used for training the GRACE-OFF models with gracemaker. GRACE_test_set.xyz: contains the 10,000 structures that were excluded from the data set prior to training and validation of the GRACE-OFF models. These structures were not used during GRACE training or validation. biased_test_set.xyz: contains 10,000 structures randomly sampled from SPICE_V2.0_cleaned.xyz. These structures may have been included in the training or validation data of both the GRACE-OFF and MACE-OFF models.

## Footnotes

1
https://github.com/openmm/spice-dataset/tree/main/downloader

## Notes

Detailed description of the energy, force, and torsion scan analysis; energy and force MAE/RMSE values on the *GRACE test set* and on 10,000 randomly selected structures (Figure S3 and Tables S3 and S4); computational performance benchmarks (ns/day) for GRACE, MACE, and UMA models across five NVIDIA GPUs (Figure S5); thermodynamic properties from NPT simulations of liquid water, benzene, methanol, acetone, *n*-hexane, and *N*-methylacetamide (NMA), including enthalpy of vaporization, heat capacity, compressibility, thermal expansion coefficient, and density (Tables S5–S9); self-diffusion coefficients for selected liquids (Table S11); and temperature-dependent density of water (Table S12) (PDF)
