# Supplementary material for: GRACE-OFF: A Machine-Learned Interatomic Potential for Organic Liquids Using the GRACE Architecture
Source: J Chem Theory Comput. 2026 Sep 3;22(18):9274–89. doi: 10.1021/acs.jctc.6c01169 (PMC13602931; doi:10.1021/acs.jctc.6c01169)
Supplement: Supplementary file 1 [file ct6c01169_si_001.pdf]

GRACE-OFF: A machine-learned interatomic potential for organic liquids using the GRACE architecture – Supporting Information

Anna Katharina Picha,[†,‡] Johannes Karwounopoulos,[*,¶] Linus C. Erhard,[¶,§] Stefan Boresch,[†] and Esther Heid[*,¶]

†*University of Vienna, Faculty of Chemistry, Institute of Computational Biological Chemistry, 1090 Vienna, Austria*

‡*University of Vienna, Vienna Doctoral School of Chemistry (DosChem), 1090 Vienna, Austria*

¶*Institute of Materials Chemistry, TU Wien, A-1060 Vienna, Austria*

§*Interdisciplinary Centre for Advanced Materials Simulation (ICAMS), Ruhr-University Bochum, 44780 Bochum, Germany*

E-mail: johannes.karwounopoulos@tuwien.ac.at; esther.heid@tuwien.ac.at

Validation strategies – Details

Error metrics for energies and forces

In this Section, we provide the detailed expressions for the energy and force prediction error metrics (see Section 3.1 in the main manuscript). Error metrics for energies and forces were computed for N_{struc} structures. For each structure $i \in \{1, \ldots, N_{\text{struc}}\}$, the reference method provides a total energy E_i^{ref} and atomic force vectors $\mathbf{F}_{i,a}^{\text{ref}}$ for every atom $a = 1, \ldots, N_i$, where

N_i is the number of atoms in structure i. Model predictions are denoted as E_i^{pred} and $\mathbf{F}_{i,a}^{\text{pred}}$.

Energy errors per atom: Energy prediction errors were calculated by taking the difference between the predicted energy E_i^{pred} and the corresponding reference energy E_i^{ref}. To obtain a size-independent metric, the total deviation for each structure was normalized by the number of atoms in the corresponding sample:

$$\varepsilon_i^{(E)} = \frac{E_i^{\text{pred}} - E_i^{\text{ref}}}{N_i}.$$

The overall mean absolute error (MAE) and root mean squared error (RMSE) per atom were then computed as

$$\text{MAE}_E = \frac{1}{N_{\text{struc}}} \sum_{i=1}^{N_{\text{struc}}} \left| \varepsilon_i^{(E)} \right|,$$

$$\text{RMSE}_E = \sqrt{\frac{1}{N_{\text{struc}}} \sum_{i=1}^{N_{\text{struc}}} \left(\varepsilon_i^{(E)} \right)^2}.$$

These quantities are reported in units of energy per atom (e.g., meV/atom).

Force errors per atom: For each structure i, atom a, and Cartesian component $\alpha \in \{x, y, z\}$, we define the force component error

$$\Delta F_{i,a,\alpha} = F_{i,a,\alpha}^{\text{pred}} - F_{i,a,\alpha}^{\text{ref}}.$$

Let the total number of atoms in the test set be

$$N_{\text{atoms}} = \sum_{i=1}^{N_{\text{struc}}} N_i.$$

The MAE and RMSE of the force components averaged over all atoms and all Cartesian directions are then

$$\text{MAE}_F = \frac{1}{3N_{\text{atoms}}} \sum_{i=1}^{N_{\text{struc}}} \sum_{a=1}^{N_i} \sum_{\alpha \in \{x,y,z\}} \left| \Delta F_{i,a,\alpha} \right|,$$

$$\mathrm{RMSE}_F = \sqrt{\frac{1}{3N_{\mathrm{atoms}}} \sum_{i=1}^{N_{\mathrm{struc}}} \sum_{a=1}^{N_i} \sum_{\alpha \in \{x,y,z\}} (\Delta F_{i,a,\alpha})^2}.$$

These metrics are reported in units of energy per distance (e.g., meV/Å).

Torsion Scans

In this Section, we describe the detailed methodology on how the error metrics for the torsion scan analysis were computed (see Section 3.3 in the main manuscript). This covers the mathematical formulation for (1) the mean cumulative area between two curves $\overline{\Delta AUC}$, (2) the mean barrier height error $\overline{\Delta BH}$, and (3) the mean absolute barrier height error $\overline{\Delta BH_{\mathrm{abs}}}$. For illustration, see Figure 1 in the main manuscript.

Let ϕ denote a single torsional (rotational) degree of freedom. For each system, we compute the MLIP energy prediction

$$E^{\mathrm{pred}}(\phi), \quad \phi \in [0, 360°],$$

by rigidly scanning the molecular geometry while varying only the torsional angle ϕ. Each scan thus yields a function $(\phi, E^{\mathrm{pred}}(\phi))$ over the interval $[0, 360°]$. We compare this predicted torsional profile to the corresponding experimental reference, denoted $E^{\mathrm{ref}}(\phi)$ as follows:

Area under the curve (AUC): The cumulative area between the predicted and the reference curve is computed separately for each torsion scan in the TorsionNet dataset[1] and referred to as ΔAUC = Delta *area under the curve*:

$$\Delta AUC = \int_0^{360} \left| E^{\mathrm{pred}}(\phi) - E^{\mathrm{ref}}(\phi) \right| d\phi.$$

The integral was evaluated using the trapezoidal rule using 24 equidistant grid points. $\overline{\Delta AUC}$ corresponds to the mean over all AUC errors, hence,

$$\overline{\Delta AUC} = \frac{1}{N_{\text{samples}}} \sum_{i=1}^{N_{\text{samples}}} \Delta AUC_i, \tag{S1}$$

where N_{samples} denotes the number of torsion profiles used for this analysis and ΔAUC_i denotes the ΔAUC error for sample $i \in \{1, \ldots, N_{\text{samples}}\}$.

Barrier height (BH): We refer to the barrier height BH as the difference between the absolute minimum and absolute maximum of one single torsion scan $E(\phi)$:

$$BH = \max_{\phi \in [0, 360°]} (E(\phi)) - \min_{\phi \in [0, 360°]} (E(\phi)).$$

We computed BH for the predicted torsional profile $E^{\text{pred}}(\phi))$, denoted as BH^{pred}, and then determine the deviation from the corresponding reference barrier height BH^{ref}, computed from the reference torsional profile $E^{\text{ref}}(\phi))$:

$$\Delta BH = BH^{\text{pred}} - BH^{\text{ref}}.$$

Correspondingly, we computed the mean over all BH errors as

$$\overline{\Delta BH} = \frac{1}{N_{\text{samples}}} \sum_{i=1}^{N_{\text{samples}}} \Delta BH_i, \tag{S2}$$

where ΔBH_i denotes the barrier height error for sample $i \in \{1, \ldots, N_{\text{samples}}\}$. Note that individual ΔBH_i error contributions can be either positive or negative. Hence, negative errors can cancel out positive errors, and vice versa. $\overline{\Delta BH} < 0$ means that, on average, the model underestimates the barrier height, $\overline{\Delta BH} > 0$ means that, on average, the model overestimates the barrier height. $\overline{\Delta BH_{\text{abs}}}$ corresponds to the mean over the absolute BH errors,

$$\overline{\Delta BH_{\text{abs}}} = \frac{1}{N_{\text{samples}}} \sum_{i=1}^{N_{\text{samples}}} |\Delta BH_i|. \tag{S3}$$

Figures

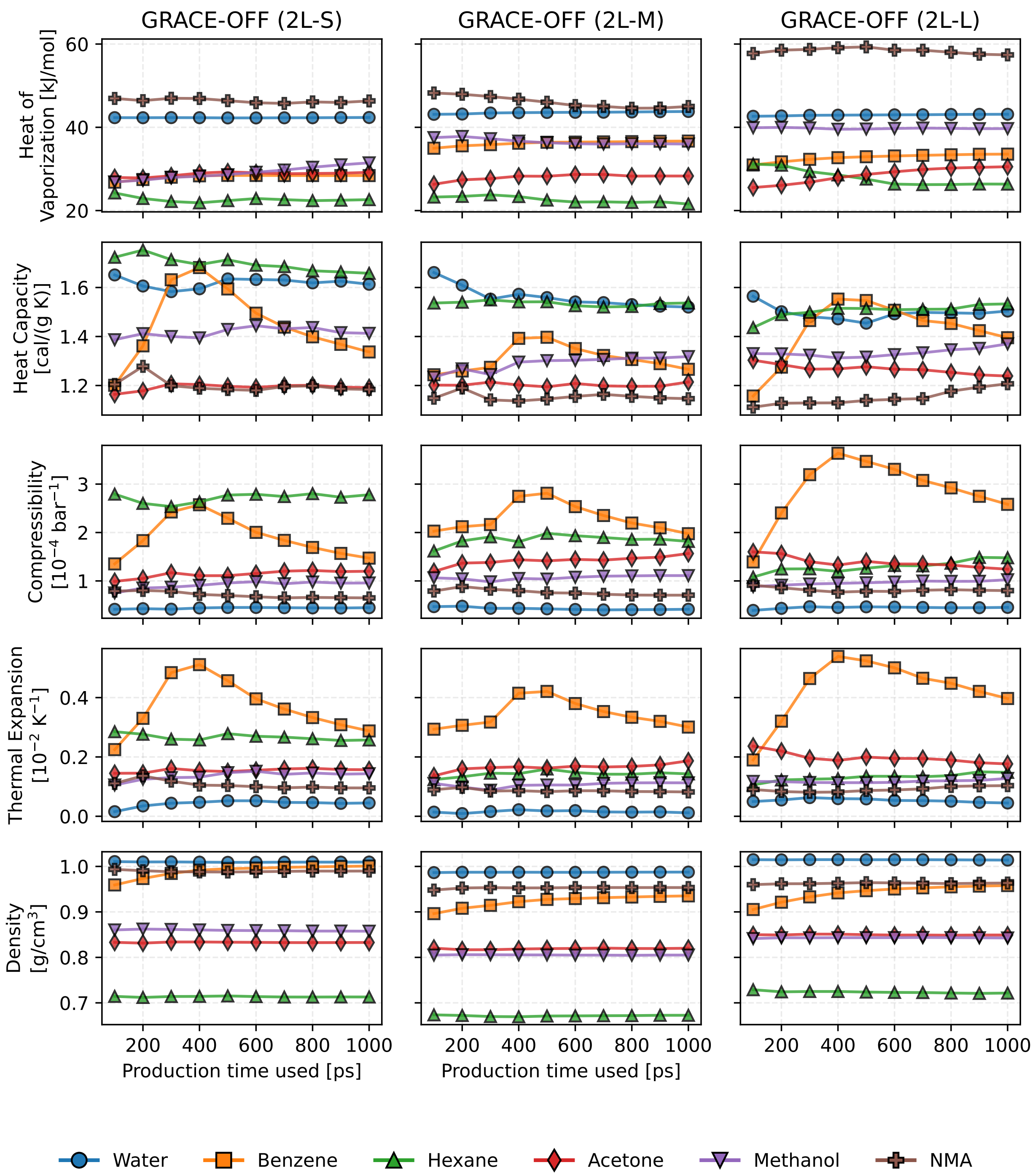

Figure S1: Convergence of condensed-phase properties as a function of simulation length. Cumulative estimates of the heat of vaporization, isothermal compressibility, thermal expansion coefficient, and density are shown for six liquids using the GRACE-OFF (2L-S/M/L) models. The first 100 ps of a 1.1 ns long simulation were discarded as equilibration, and the properties were calculated using progressively longer production intervals of up to 1 ns.

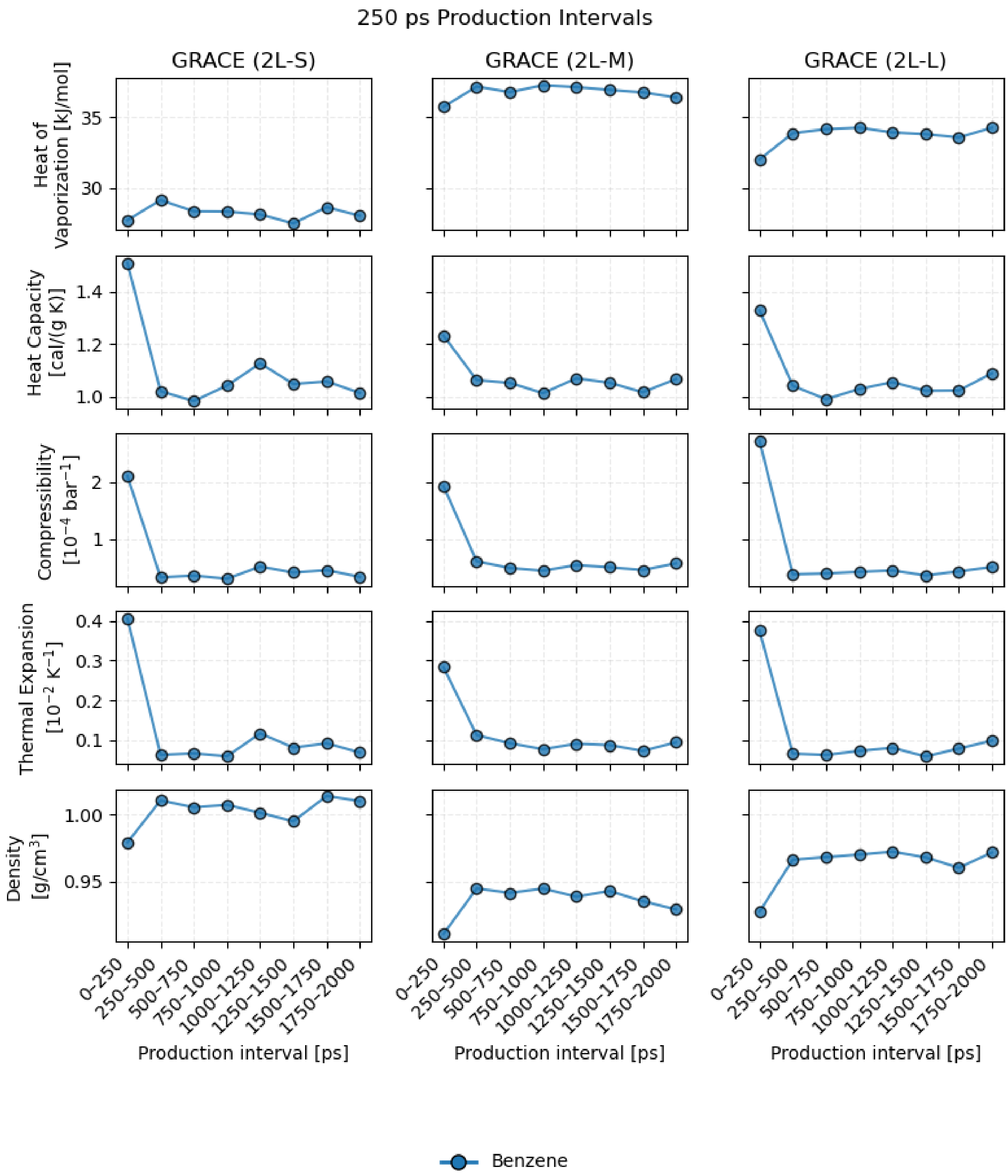

Figure S2: Convergence of the investigated physical properties for benzene computed from an independent, additional 2.2 ns simulation. Properties were calculated over disjoint 200 ps intervals, the first 250 ps were discarded as equilibration.

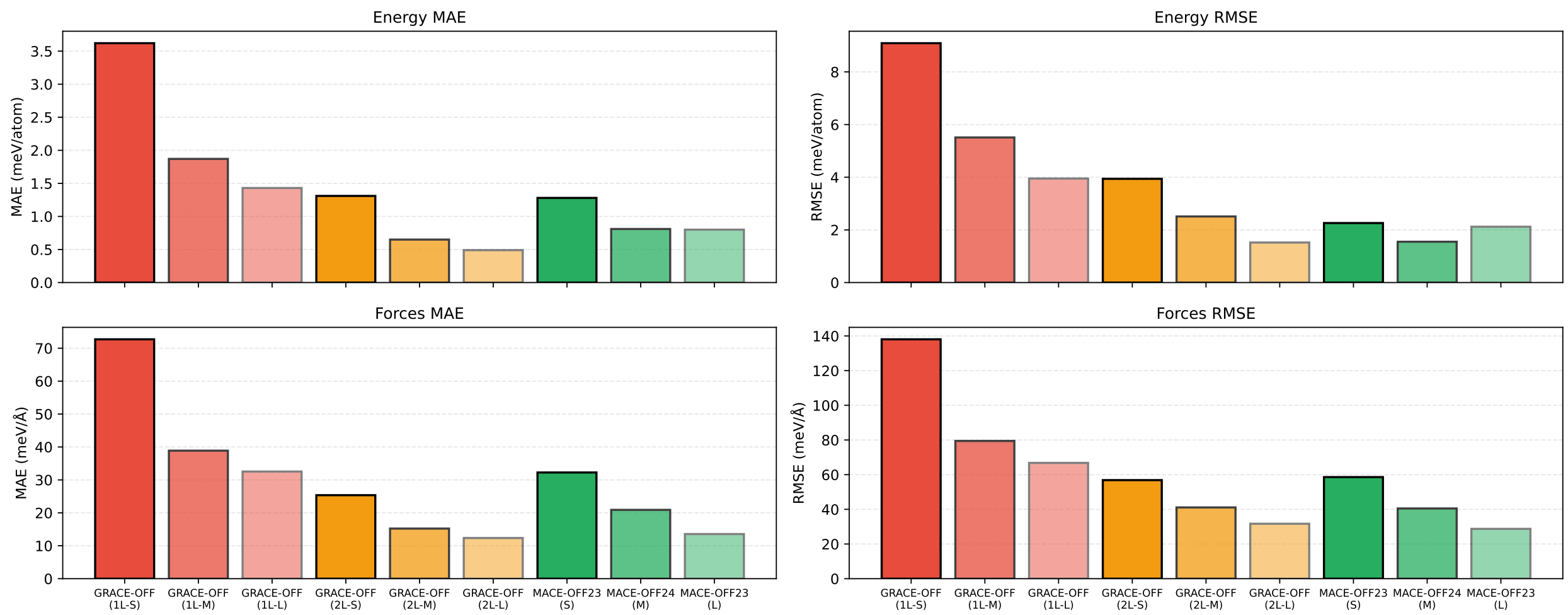

Figure S3: Energy MAE and RMSE as well as force MAE and RMSE per atom are shown for all GRACE-OFF models (one- and two-layer architectures) across all model sizes (small, medium, large), in comparison to the corresponding MACE models. The evaluation was performed on 10,000 structures selected prior to the training–validation split, ensuring that they were not seen by the GRACE-OFF models during training. For the MACE models, it is unknown whether these structures were included in their training or validation sets (*GRACE test set*).

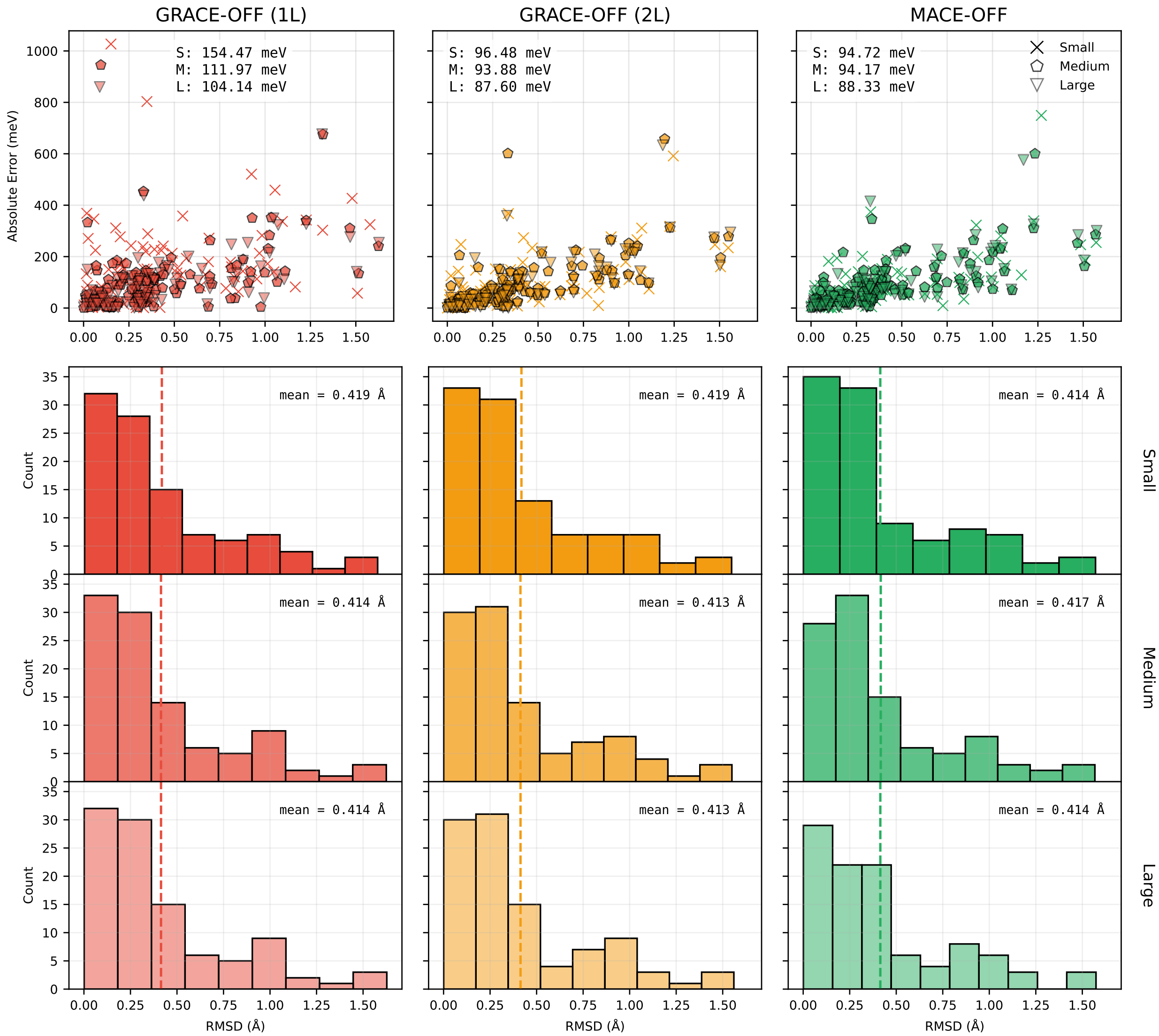

Figure S4: Results for 100 geometry optimized structures taken from the *GRACE test set*. Upper panel: The deviation of the predicted energy from the QM optimized energy per molecule is shown on the y-axis, while the x-axis represents the root-mean-square deviation (RMSD) between the GRACE-OFF/MACE-OFF optimized structures and the corresponding QM-optimized geometries. From left to right, results are shown for the one-layer GRACE-OFF models, the two-layer GRACE-OFF models, and the MACE-OFF models, each evaluated for the small, medium, and large model sizes. The lower panel shows the corresponding RMSD distributions across molecules relative to the QM-optimized geometries, with the dashed line highlighting the mean absolute error.

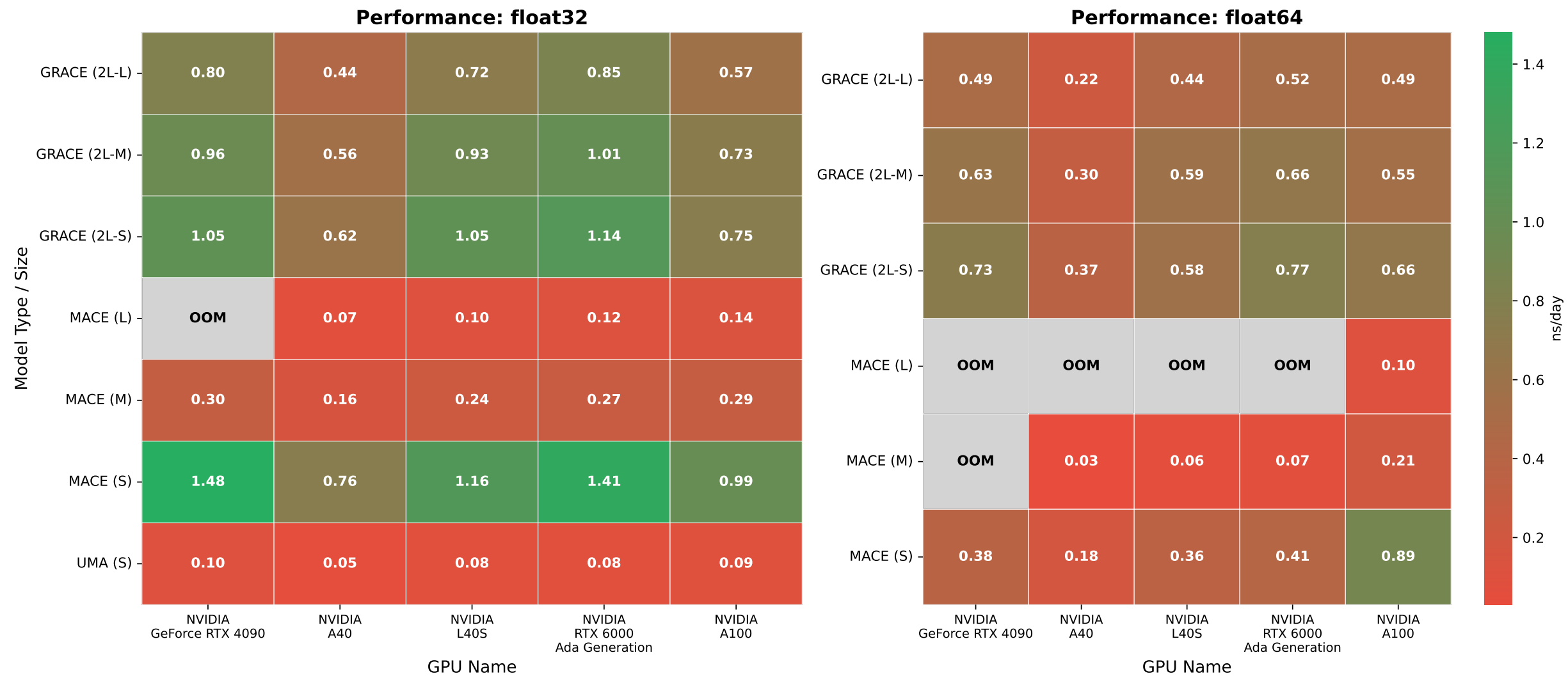

Figure S5: Computational performance in ns/day for different GRACE, MACE, and UMA models across several NVIDIA GPUs. Results are shown for single-precision (float32) in the left matrix and double-precision (float64) simulations in the right matrix. We show results for the performance on a NVIDIA GeForce RTX 4090, NVIDIA A40, NVIDIA L40S, NVIDIA RTX 6000 Ada, and NVIDIA A100. If a model could not be run, this is indicated by the label OOM (out of memory).

Additional Tables

Comparison of ASE and OpenMM literature results

To check that the use of the ASE package rather than OpenMM (as in Ref. 2) has no systematic influence on the results, we calculated water properties from an MD simulation using ASE and compared the results with literature data obtained from simulations performed with OpenMM as the MD engine. The OpenMM results are the mean obtained from five repeats (see SI of Ref. 2); the deviations from the ASE results are commensurate with the statistical error.

Table S1: Thermodynamic properties of water obtained from NPT simulations using either ASE or OpenMM as MD engine for the MACE-OFF23 (S)model.

MD engine	ΔH_{vap} [kJ/mol]	C_p [cal/(g K)]	κ [10^{-4}/bar]	α [10^{-2}/K]	ρ [g/mL]
Experiment	43.99	1.00	0.45	0.03	0.997
ASE	47.74 ± 0.04	1.68 ± 0.04	0.28 ± 0.01	0.10 ± 0.00	1.11 ± 0.00
OpenMM[a]	47.80 ± 0.02	1.71 ± 0.07	0.29 ± 0.01	0.10 ± 0.01	1.12 ± 0.00

[a] Values from Ref 2.

Training parameters

Table S2: Input parameters used for training the GRACE-OFF models for the different number of layers (1L and 2L) and model sizes (small/medium/large). If a parameter has the same value for all six models, the value is shown once in a centered merged cell spanning all model columns. For training GRACEMAKER version 0.5.1 was used (`https://github.com/ICAMS/grace-tensorpotential/releases/tag/0.5.1`.

	Small		Medium		Large	
**Parameter**	**1L**	**2L**	**1L**	**2L**	**1L**	**2L**
**Model architecture**						
GRACE preset	`GRACE_1LAYER`	`GRACE_2LAYER`	`GRACE_1LAYER`	`GRACE_2LAYER`	`GRACE_1LAYER`	`GRACE_2LAYER`
Cutoff radius, r_{cut} (Å)	6.0					
Maximum angular momentum, $l_{\max}$	3	$[3, 2]$	4	$[3, 3]$	4	$[4, 3]$
Maximum number of radial channels, $n_{\mathrm{rad,max}}$	20	$[20, 32]$	32	$[32, 42]$	48	$[32, 48]$
Maximum product order	3	3	4	4	4	4
Number of nonlinear readout densities ($n_{\mathrm{MLP,dens}}$)	10	8	12	10	16	12
GRACEmaker validation fraction	0.05					
Reference-energy correction	0					
Random seed	1					
**Energy and force loss**						
Energy-loss type	Huber					
Force-loss type	Huber					
Initial energy-loss weight	1					
Initial force-loss weight	5					
Energy Huber threshold, δ_E	0.001					
Force Huber threshold, δ_F	0.001					
Loss-switch epoch	300					
Energy-loss weight after switching	5					
Force-loss weight after switching	2					
Learning rate after loss switch	0.001					
**Optimization**						
Optimizer	Adam					
Maximum number of epochs, `maxiter`	500					
Initial learning rate	1.0×10^{-2}					
AMSGrad	Enabled					
Exponential moving average	Enabled					
EMA momentum	0.99					
Weight decay	None					
Gradient clipping value	1.0					
**Learning-rate reduction**						
Scheduler	Reduction on plateau					

Continued on next page

Table S2 – continued

Parameter	Small 1L	Small 2L	Medium 1L	Medium 2L	Large 1L	Large 2L
Patience	5 epochs					
Reduction factor	0.98					
Minimum learning rate	5.0×10^{-4}					
Stop at minimum learning rate	Enabled					
Resume stored learning rate	Enabled					
**Batching and data processing**						
Training batch size	32					
Validation batch size	64					
Maximum number of training buckets	100					
Maximum number of validation buckets	50					
Shuffle training data	Enabled					
Compute convex hull	Disabled					
**Implementation and output**						
JIT compilation	Enabled					
Evaluate initial statistics	Disabled					
Checkpoint frequency (epochs)	50					
Display progress bar	Enabled					

Thermodynamic properties and diffusion coefficients

Table S3: Model performance metrics for the *GRACE test set*. Energies in meV; forces in meV/Å. The corresponding plot can be seen in Figure S3.

Model	Energy RMSE (meV)	Energy MAE (meV)	Forces RMSE (meV/Å)	Forces MAE (meV/Å)
GRACE-OFF (1L-S)	9.09	3.62	138.0723	72.7009
GRACE-OFF (1L-M)	5.51	1.87	79.4485	38.8797
GRACE-OFF (1L-L)	3.95	1.43	66.7743	32.5390
GRACE-OFF (2L-S)	3.94	1.31	56.8564	25.3582
GRACE-OFF (2L-M)	2.51	0.65	41.0955	15.2176
GRACE-OFF (2L-L)	1.52	0.49	31.6786	12.3554
MACE-OFF23 (S)	2.26	1.28	58.6003	32.2563
MACE-OFF24 (M)	1.55	0.81	40.5053	20.9003
MACE-OFF23 (L)	2.12	0.80	28.7381	13.5563

Table S4: Model performance metrics for the *biased test set*. Energies in meV; forces in meV/Å for the 10,000 randomly chosen structures. The data listed here are shown in Figure 1 in the main manuscript.

Model	Energy RMSE (meV)	Energy MAE (meV)	Forces RMSE (meV/Å)	Forces MAE (meV/Å)
GRACE-OFF (1L-S)	8.67	3.46	138.7688	71.4809
GRACE-OFF (1L-M)	4.27	1.78	81.1765	38.5286
GRACE-OFF (1L-L)	3.82	1.38	67.2768	32.0414
GRACE-OFF (2L-S)	2.98	1.21	57.4230	24.8826
GRACE-OFF (2L-M)	2.01	0.62	38.6641	14.8365
GRACE-OFF (2L-L)	1.95	0.47	34.1059	12.0665
MACE-OFF23 (S)	2.54	1.26	64.0469	31.9850
MACE-OFF24 (M)	1.87	0.81	47.7470	20.6761
MACE-OFF23 (L)	2.73	0.79	41.4044	13.6198

Table S5: Thermodynamic properties of liquid **water** obtained from NPT simulations using selected GRACE-OFF and MACE-OFF models, as well as UMA/OMol25 (S), compared to experimental values.

Model	ΔH_{vap} [kJ/mol]	C_p [cal/(g K)]	κ [10^{-4}/bar]	α [10^{-2}/K]	ρ [g/mL]
Experiment	43.99	1.00	0.45	0.03	0.997
GRACE-OFF (2L-S)	42.36	1.61	0.45	0.05	1.01
GRACE-OFF (2L-M)	43.31	1.58	0.42	0.01	1.00
GRACE-OFF (2L-L)	43.10	1.50	0.46	0.05	1.01
MACE-OFF23 (S)[a]	47.80	1.71	0.29	0.10	1.12
MACE-OFF24 (M)	47.47	1.42	0.41	0.08	1.08
MACE-OFF23 (M)[a]	48.90	1.57	0.23	0.06	1.18
UMA/OMol25 (S)	50.38	1.54	0.34	0.07	1.11

[a] Values from Ref 2.

Table S6: Thermodynamic properties of liquid **benzene** obtained from NPT simulations using selected GRACE-OFF and MACE-OFF models compared to experimental values. For the GRACE-OFF models, all properties were computed from two separate simulations: The final ns of the original 1.1 ns simulation and the final ns of the additional 2.2 ns simulation (denoted with a *). A convergence analysis of the additional 2.2 ns simulation is shown in Figure S2.

Model	ΔH_{vap} [kJ/mol]	C_p [cal/(g K)]	κ [10^{-4}/bar]	α [10^{-2}/K]	ρ [g/mL]
Experiment	33.83	0.42	0.97	0.11	0.88
GRACE-OFF (2L-S)	27.86	1.09	0.52	0.11	1.00
GRACE-OFF (2L-S)*	28.04	1.13	0.63	0.13	1.01
GRACE-OFF (2L-M)	37.06	1.08	0.70	0.12	0.96
GRACE-OFF (2L-M)*	36.72	1.07	0.69	0.11	0.93
GRACE-OFF (2L-L)	31.18	1.05	0.49	0.09	0.96
GRACE-OFF (2L-L)*	33.88	1.07	0.54	0.1	0.96
MACE-OFF23 (S)[a]	36.19	1.18	0.72	0.15	1.02
MACE-OFF23 (M)[a]	28.11	1.24	1.97	0.27	0.89

[a] Values from Ref 2.

Table S7: Thermodynamic properties of liquid **methanol** obtained from NPT simulations using selected GRACE-OFF and MACE-OFF models compared to experimental values.

Model	ΔH_{vap} [kJ/mol]	C_p [cal/(g K)]	κ [10^{-4}/bar]	α [10^{-2}/K]	ρ [g/mL]
Experiment	38.30	0.61	0.31	0.15	0.79
GRACE-OFF (2L-S)	31.06	1.42	0.98	0.15	0.864
GRACE-OFF (2L-M)	35.98	1.32	1.11	0.11	0.814
GRACE-OFF (2L-L)	39.65	1.38	1.04	0.13	0.829
MACE-OFF23 (S)[a]	36.29	1.43	1.52	0.21	0.91
MACE-OFF23 (M)[a]	34.98	1.40	2.30	0.20	0.81

[a] Values from Ref 2.

Table S8: Thermodynamic properties of liquid **acetone** obtained from NPT simulations using selected GRACE-OFF and MACE-OFF models compared to experimental values.

Model	ΔH_{vap} [kJ/mol]	C_p [cal/(g K)]	κ [10^{-4}/bar]	α [10^{-2}/K]	ρ [g/mL]
Experiment	31.90	0.52	1.41	0.15	0.78
GRACE-OFF (2L-S)	28.85	1.19	1.23	0.16	0.84
GRACE-OFF (2L-M)	28.35	1.21	1.62	0.19	0.812
GRACE-OFF (2L-L)	30.23	1.23	1.20	0.17	0.833
MACE-OFF23 (S)[a]	29.93	1.25	1.24	0.17	0.88
MACE-OFF23 (M)[a]	20.05	1.23	29.09	1.10	0.64

[a] Values from Ref 2.

Table S9: Thermodynamic properties of liquid ***n*-hexane** obtained from NPT simulations using selected GRACE-OFF and MACE-OFF models, as well as UMA/OMol25, compared to experimental values.

Model	ΔH_{vap} [kJ/mol]	C_p [cal/(g K)]	κ [10^{-4}/bar]	α [10^{-2}/K]	ρ [g/mL]
Experiment	31.52	0.54	1.67	0.14	0.66
GRACE-OFF (2L-S)	22.66	1.64	2.79	0.25	0.68
GRACE-OFF (2L-M)	24.39	1.53	1.78	0.14	0.652
GRACE-OFF (2L-L)	26.57	1.54	1.47	0.15	0.724
MACE-OFF23 (S)[a]	33.10	2.19	3.79	0.53	0.79
MACE-OFF24 (M)	15.28	1.66	14.11	0.53	0.55
MACE-OFF23 (M)[a]	15.77	2.12	327.35	3.44	0.39
UMA/OMol25 (S)	33.82	1.58	0.84	0.12	0.74

[a] Values from Ref 2.

Table S10: Thermodynamic properties of liquid **N-methylacetamide (NMA)** obtained from NPT simulations using selected GRACE-OFF and MACE-OFF models compared to experimental values.

Model	ΔH_{vap} [kJ/mol]	C_p [cal/(g K)]	κ [10^{-4}/bar]	α [10^{-2}/K]	ρ [g/mL]
Experiment	53.50	–	–	–	0.92
GRACE-OFF (2L-S)	58.23	1.25	0.66	0.11	0.973
GRACE-OFF (2L-M)	45.27	1.16	0.77	0.10	0.973
GRACE-OFF (2L-L)	57.62	1.21	0.78	0.11	0.948
MACE-OFF23 (S)[a]	58.83	1.27	0.48	0.11	1.07
MACE-OFF23 (M)[a]	46.82	1.11	1.87	0.14	0.87

[a] Values from Ref 2.

Table S11: Self-diffusion coefficients D ($\cdot 10^{-9}$ m^2/s) for five liquids for selected GRACE-OFF and MACE-OFF models. The calculated value with the best agreement to the experimental data is highlighted in boldface.

Model	Water	Methanol	Acetone	Benzene	*n*-Hexane
Experiment	2.29	2.27	4.94	2.15	4,14
GRACE-OFF (2L-S)	2.18	2.83	4.47	**2.00**	3.49
GRACE-OFF (2L-M)	2.04	**2.41**	**4.49**	3.16	**3.87**
MACE-OFF23(S)	**2.20**	57.50	1.14	0.21	0.10

Table S12: Mean density of a pure waterbox at various temperatures computed from 100ps NPT simulations for selected GRACE-OFF and MACE-OFF models.

Temperature [K]	270	280	290	300	310	320	330
Experiment	–	1.00	1.00	1.00	0.99	0.99	0.98
GRACE-OFF (2L-S)	1.02	1.02	1.02	1.01	1.01	1.00	0.99
GRACE-OFF (2L-M)	0.99	0.99	0.99	0.99	0.99	0.99	0.98
GRACE-OFF (2L-L)	1.03	1.02	1.02	1.02	1.01	1.01	0.99
MACE-OFF23 (S)	1.15	1.14	1.12	1.11	1.10	1.09	1.08
MACE-OFF24 (M)	1.10	1.09	1.08	1.07	1.06	1.06	1.05

References

(1) Rai, B. K.; Sresht, V.; Yang, Q.; Unwalla, R.; Tu, M.; Mathiowetz, A. M.; Bakken, G. A. TorsionNet: A Deep Neural Network to Rapidly Predict Small-Molecule Torsional Energy Profiles with the Accuracy of Quantum Mechanics. *J. Chem. Inf. Model.* **2022**, *62*, 785–800.

(2) Picha, A.; Wieder, M.; Boresch, S. Transferable neural network potentials and condensed phase properties. *J. Chem. Inf. Model.* **2025**, *65*, 9483–9496.
